# Supplementary material for: Reconstructing the evolution history of networked complex systems
Source: Nat Commun. 2024 Apr 2;15:2849. doi: 10.1038/s41467-024-47248-x (PMC10987487; doi:10.1038/s41467-024-47248-x)
Supplement: Supplementary file 1 — Supplementary Information [file 41467_2024_47248_MOESM1_ESM.pdf]

Reconstructing the evolution history of networked complex systems–Supplementary Information

Junya Wang[1], Yi-Jiao Zhang[2], Cong Xu[2], Jiaze Li[3], Jiachen Sun[4], Jiarong Xie[5, 6], Ling Feng[7, 8], Tianshou Zhou[9], and Yanqing Hu*[2,10]

[1]School of Systems Science and Engineering, Sun Yat-sen University, Guangzhou, 510006, China
[2]Department of Statistics and Data Science, College of Science, Southern University of Science and Technology, Shenzhen, 518055, China
[3]Department of Data Analytics and Digitalisation, School of Business and Economics, Maastricht University, Maastricht, 6200MD, The Netherlands
[4]Tencent Inc. Shenzhen, 518000, China
[5]Center for Computational Communication Research, Beijing Normal University, Zhuhai, 519087, China
[6]School of Journalism and Communication, Beijing Normal University, Beijing, 100875, China
[7]Institute of High Performance Computing (IHPC), Agency for Science, Technology and Research (A*STAR), 138632, Singapore
[8]Department of Physics, National University of Singapore, 117551, Singapore
[9]School of Mathematics, Sun Yat-sen University, Guangzhou, 510275, China
[10]Center for Complex Flows and Soft Matter Research, Southern University of Science and Technology, Shenzhen, 518055, China

March 13, 2024

Contents

1 Machine learning algorithm **3**
1.1 Edge Representation methods 4
1.2 CPNN model 8
1.2.1 Architecture of CPNN 8
1.2.2 Loss Function of CPNN 9

*yanqing.hu.sc@qq.com

1.2.3 Training process of CPNN . . . 10
1.3 Ensemble model . . . 10
1.4 Construction of Training set and Test set . . . 11

2 Ranking methods **12**

3 Data Set **13**
3.1 Protein-protein interaction (PPI) networks . . . 13
3.2 World trade web . . . 15
3.3 Collaboration networks . . . 15
3.4 Animal networks . . . 15
3.5 Transportation networks . . . 16

4 Edge Restoration results on real-world networks. **16**

5 Accuracy of the machine learning algorithm to predict generation order of edge pairs **18**
5.1 Accuracy of different methods on different real-world networks . . . 18
5.2 Influence of training ratio on the prediction accuracy . . . 19
5.3 Accuracy of CPNN model with single edge index . . . 19
5.4 Effect of coarse-grained edge generation time on the edge pair prediction accuracy . . . 21

6 Overall error of the restored edge sequence **22**
6.1 Theoretical relation between the prediction accuracy of two edges' generation order and the overall error $\mathcal{E}$ . . . 22
6.2 Equivalence between overall error $\mathcal{E}$ and other correlation coefficients . . . 26
6.2.1 Theoretical equivalence between $\mathcal{E}$ and Kendall's τ. . . . 26
6.2.2 Theoretical equivalence between $\mathcal{E}$ and Spearman's ρ. . . . 27

7 Discussion of restoration results for networks lacking ground truth **27**

8 Transfer Learning **31**
8.1 Synthetic network models . . . 31
8.2 Vector transformation of nodes in transfer learning . . . 32

9 Revealing the evolution mechanisms by the restored edge sequence **34**
9.1 Preferential attachment in real-world networks . . . 34
9.2 Protein categories: the abbreviations and the corresponding functions . . . 35
9.3 Methods to generate network based on preferential attachment (PA) rule . . . 36
9.4 Other network characterizations revealed by the restored edge sequence . . . 39

10 Link prediction with restored edge sequence **43**
10.1 Detailed information of removed edges for link prediction 43
10.2 Facilitating the results of common neighbor for link prediction 43
10.3 Facilitating the results of Adamic-Adar index for link prediction 44
10.4 Facilitating the results of structural perturbation method (SPM) for link prediction . . . 46

Supplementary References **49**

1 Machine learning algorithm

In this section, we introduce how to discriminate the generation order between two given edges by machine learning techniques. The schematic illustration of the proposed framework is shown in Fig. S1. We first use edge representation methods to convert each edge in the network into a real-value vector, then employ a comparative paradigm-based neural network (CPNN) [1] to learn the generation order information of a pair of edges (i.e., to tell which edge is added earlier). In this work, six different edge representation methods are used, resulting in six trained CPNN models, which are denoted as the base models. In addition, we calculate 11 indexes of edges, directly compare the accuracy of these 11 indexes for edge generation order prediction, and select the one with the highest accuracy as the ”best feature”. The prediction results of the best feature and the six CPNN base models are together fed into an ensemble learning model, which outputs the ultimate prediction of generation order between two edges.

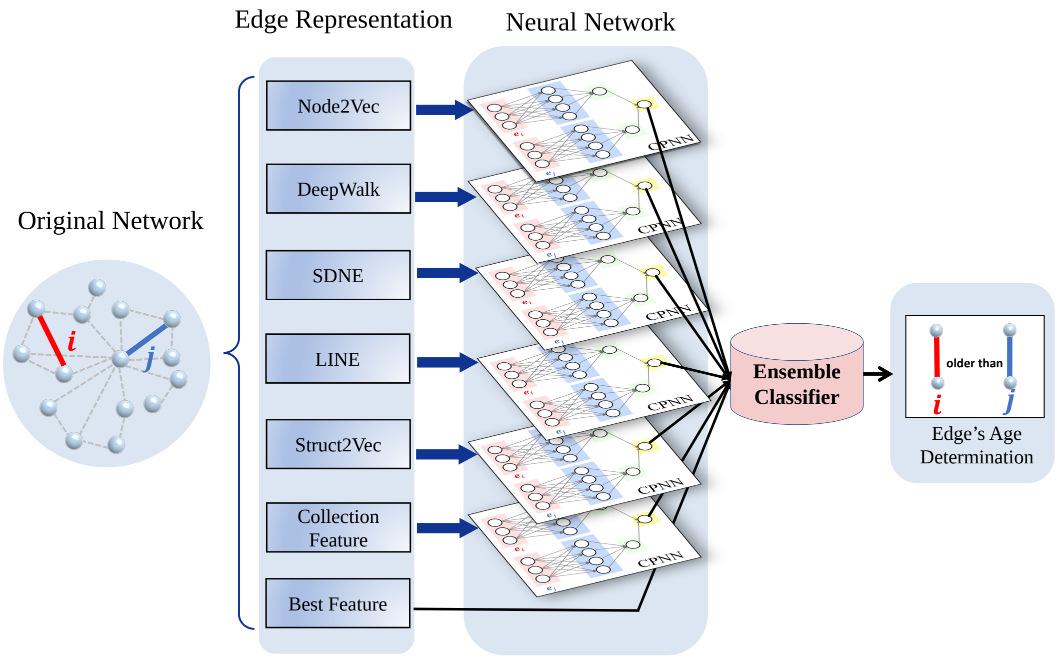

Figure S1: **Illustration of the Ensemble model to predict the generation order of two edges.** First, use different edge representation methods to convert two edges e_1 and e_2 into vectors. Then use the CPNN model to get the edge generation order prediction results based on different edge representations. Finally, the ensemble model is employed to combine the results of each CPNN base model and of the best feature to get the optimal prediction. Note that the value of the best feature can be fed into the ensemble model directly without being trained by CPNN model.

Next, we introduce details of the edge representation methods; the architecture, loss function and training process of the CPNN model; the parameters training method of the ensemble model; and the rule to split training set and test set.

1.1 Edge Representation methods

Here we consider two different types of edge representation techniques. The first one is network embedding learning, which learns low-dimensional representations of nodes or edges according to the connection relationship in the network. In this work, we first employ the widely-used node representation methods to get the low-dimensional vectors of nodes. Then, we get the vector representation of the corresponding edge by calculating the Hadamard product of the vectors of two nodes. Suppose the two ends of edge i are node i_1 and node i_2, $\mathbf{e}_i^d$, $\mathbf{e}_{i_1}^d$ and $\mathbf{e}_{i_2}^d$ are the d-dimensional representations

of edge i and node i_1, i_2, then we have

$$\mathbf{e}_i^d = \mathbf{e}_{i_1}^d \circ \mathbf{e}_{i_2}^d. \tag{S1}$$

Regarding the node representation, we consider five widely-used embedding methods, including Deepwalk [2], Node2Vec [3], LINE [4], Struc2Vec [5], and SDNE [6]. All the embedding algorithms are implemented using the code published by the authors of the corresponding embeddings. A brief introduction of the five embedding methods is as follows.

(1) DeepWalk [2]. Deepwalk generates multiple fixed-length node sequences through random walks, and then learns a vector representation that maximizes the co-occurrence of nodes on the sequences. The parameters we use in this work are set to: number of walks = 80, walk length = 10, window size = 5, and the dimension of output vector $d = 128$.

(2) Node2Vec [3]. Node2Vec is a node representation learning method similar to DeepWalk. Node2Vec is also based on a random walk, but considers both depth-first sampling and breadth-first sampling by adding biased parameters p and q. The parameters we use are: number of walks = 10, walk length = 80, window size = 10, dimension = 128.

(3) LINE [4]. LINE is an embedding method that preserves both first-order similarity and second-order similarity among nodes. The first-order similarity between nodes a and b is the connecting edges between them, which can be measured by the joint probability distribution as follows,

$$p_1(a, b) = \frac{1}{1 + exp(-\mathbf{e}_a^T \mathbf{e}_b)}. \tag{S2}$$

The second-order similarity between nodes a and b is the common neighbor between them. It can be measured by the following conditional probability distribution:

$$p_2(b|a) = \frac{exp(\mathbf{e}_b'^T \mathbf{e}_a')}{\sum_{k=1}^{|V|} exp(\mathbf{e}_k'^T \mathbf{e}_a')}, \tag{S3}$$

where $|V|$ is the set of nodes and context nodes. For node a, $\mathbf{e}_a$ and $\mathbf{e}_a'$ are the vector representations of node a and the vector representation when node a is seen as context, respectively. The node vectors that preserve both first-order and second-order similarity can be obtained by minimizing the above two distributions and the Kullback-Leibler (KL) divergence of their corresponding real distributions. The parameters in this work are set to: dimension = 128, batch size of training = 1024, epochs = 50.

(4) Struc2Vec [5]. Struc2Vec is an embedding algorithm based on structure similarity. First, construct a new graph – context graph based on the structure similarity between nodes. The context graph is a multilayer network that keeps nodes with similar structures closer to each other. Then a biased random walk and skip-gram algorithm is used on the context graph to get the vector representations of nodes. The parameters in this work are set to: dimension = 128, number of walks = 80, walk length = 10, window size = 10, and the probability that the random walk does not change layers = 0.3.

(5) SDNE [6]. SDNE is a method based on deep learning, which uses a deep autoencoder to preserve both the first-order and second-order similarity of a network. The parameters in this work are set to: the dimension of the output vectors = 128, the dimension of the hidden layers = 256, L_1-norm regularisation parameter = $1e-5$, L_2-norm regularisation parameter = $1e-4$, batch size of training = 3000, epochs = 40.

The second type of edge representation is a combination of 11 edge indexes. In order to capture more structure information of a network, we calculate 11 popular structure indexes of edges, and put them together to form an 11-dimensional vector as the edge representation. We denote the 11-dimensional vectors as the "Collection feature" of the edges. The 11 edge indexes are introduced below.

For simplicity, we denote that i_1 and i_2 are two endpoints of edge i, A is the adjacency matrix of the network, $k(a)$ is the degree of node a, $\Gamma(a)$ is the set of neighbors of node a, $|\Gamma|$ is the cardinality of the set Γ.

(1) Edge betweenness [7]. The edge betweenness (BN) of an edge is defined as the proportion of the number of the shortest paths pass through the edge to the number of all shortest paths.

$$\mathrm{BN}(i) = \sum_{a,b \in V} \frac{\sigma(a,b|i)}{\sigma(a,b)}, \tag{S4}$$

where V is the set of nodes, $\sigma(a,b|i)$ is the number of the shortest paths pass through edge i between node a and b, $\sigma(a,b)$ is the number of the shortest path between node a and b.

(2) Edge degree. Edge degree (DEG) is defined as the sum of the degrees of the nodes at its two ends.

$$\mathrm{DEG}(i) = k(i_1) + k(i_2). \tag{S5}$$

(3) Common neighbor [8]. Common neighbor (CN) between two nodes at the ends of an edge is defined as the number of the common neighbor of the two nodes.

$$\mathrm{CN}(i) = |\Gamma(i_1) \cap \Gamma(i_2)| = (A^2)_{i_1 i_2}. \tag{S6}$$

(4) Edge clustering coefficient [9]. The edge clustering coefficient describes the clustering degree of nodes around the edge. It is defined as:

$$\mathrm{CC}(i) = \frac{|\Gamma(i_1) \cap \Gamma(i_2)|}{\min\{k(i_1)-1, k(i_2)-1\}}. \tag{S7}$$

(5) Edge strength [10]. Edge strength (STR) of edge i is defined as the proportion of the common neighbors of i's two ends i_1 and i_2 to all the neighbors of i_1 and i_2.

$$\mathrm{STR}(i) = \frac{|\Gamma(i_1) \cap \Gamma(i_2)|}{|\Gamma(i_1) \cup \Gamma(i_2)|}. \tag{S8}$$

(6) Resource allocation index [11]. Resource allocation index (RA) counts common neighbors and gives more weights to smaller degree nodes,

$$\mathrm{RA}(i) = \sum_{z\in\Gamma(i_1)\cap\Gamma(i_2)} \frac{1}{k(z)}. \tag{S9}$$

(7) Adamic-Adar index [12]. Adamic-Adar index (AA) is similar to the Resource allocation index, but with a slightly different weight calculation method,

$$\mathrm{AA}(i) = \sum_{z\in\Gamma(i_1)\cap\Gamma(i_2)} \frac{1}{\log(k(z))}. \tag{S10}$$

(8) Preferential attachment index [13, 14]. Preferential attachment index (PA) is defined as the product of the degree of two end nodes of an edge,

$$\mathrm{PA}(i) = k(i_1) \times k(i_2). \tag{S11}$$

(9) Local path index [15]. Local path index (LP) considers not only common neighbors (the number of paths with a length equal to 2 between two nodes) but also the number of paths with a length equal to 3. It defined as

$$\mathrm{LP}(i) = (A^2)_{i_1 i_2} + \epsilon(A^3)_{i_1 i_2}. \tag{S12}$$

Local path index degenerates to common neighbor index when $\epsilon = 0$. In this work, we set $\epsilon = 0.01$.

(10) Edge PageRank [16]. We define edge PageRank (PR) as the maximum value of the PageRank of the nodes at two ends of an edge,

$$\mathrm{PR}(i) = \max\left\{\mathrm{PR}(i_1), \mathrm{PR}(i_2)\right\}, \tag{S13}$$

where $\mathrm{PR}(i_1)$ and $\mathrm{PR}(i_2)$ are PageRank values of nodes i_1 and i_2.

(11) Edge k-shell [17]. K-shell decomposition is an algorithm that decomposes the network layer by layer based on the degree of nodes. First, successively remove all nodes with degree to one until there are no nodes with degree one in the network. All the removed nodes and edges are denoted as 1-shell nodes and edges. Then do the same thing for the nodes with degree two, and get the 2-shell nodes and edges. In this way, if edge i is removed in the k-shell, then the k-shell value of the edge is KS(i) = k.

In order to remove the scale effect from various dimensions, each index value needs to be normalized before being combined into an 11-dimensional vector. We use the max-min approach to normalize the value of each index. Set the maximum and minimum value of one index as $F_{\max}$ and $F_{\min}$, respectively. The value of this index for edge i is F,then the normalized value of F is

$$F_{\mathrm{norm}} = \frac{F - F_{\min}}{F_{\max} - F_{\min}}. \tag{S14}$$

In addition to the 11-dimensional "Collection feature", we also consider directly using one single index to predict the generation order of edge pairs. We calculate the accuracy of each index to discriminate the generation order of the edge pairs in the training set (randomly give a judgment when the values of the index for two edges are the same). Then the index that has the highest accuracy in the training set is selected as the "Best feature" to predict the generation order for other edge pairs. Different from the other six edge representation methods (five edge embedding methods and the collection feature), the best feature can be used by direct comparison. So the best feature is fed into the ensemble model without the CPNN training process.

1.2 CPNN model

After getting the vector representations of the edges, we need a machine learning model to determine the generation order of two edges. In order to avoid inconsistent judgment results when switching the input order of edges, we use a symmetric neural network – comparison paradigm neural network based on Ref. [1]. Next, we introduce the details of the structure, loss function, and training process of the CPNN model.

1.2.1 Architecture of CPNN

In this subsection, we give a detailed description of CPNN [1]. As illustrated in Fig. S2, the CPNN model accepts the vector representations of two edges as input, each input edge propagates forward independently to obtain an output value, which is combined together to obtain the final output. As such, CPNN is able to ensure the consistency of the output regardless of the order of the input.

Specifically, CPNN is mainly composed of a 3-layer fully connected neural network, where an input layer accepts the vector representation of an edge, a hidden layer, and a single-dimensional output layer. According to the existing research on neural network architecture [18], here we set the number of dimensions in the hidden layer as the sum of 2/3 of the input layer dimension and the output layer dimension (i.e., 1). The activation function of the hidden layer is set as the ReLU function:

$$\mathrm{Relu}(x) = \max(0, x). \tag{S15}$$

Two edges i and j propagate forward independently through the CPNN, and the output results are concentrated into a 2-dimensional vector $[z_i, z_j]$, which is then normalized by a softmax function S, resulting in the final output $[\mathrm{S_i}, \mathrm{S_j}] = \mathrm{S}([z_i, z_j])$:

$$\begin{aligned} \mathrm{S_i} &= \frac{e^{z_i}}{e^{z_i} + e^{z_j}}, \\ \mathrm{S_j} &= \frac{e^{z_j}}{e^{z_i} + e^{z_j}}, \end{aligned} \tag{S16}$$

where e represents the base of the natural logarithm.

Overall, the structure of CPNN can be formalized as follows:

$$\begin{aligned} \mathbf{O} &= \mathrm{S}([z_i, z_j]), \\ z_i &= \mathbf{W}_2 \mathrm{Relu}(\mathbf{W}_1 \mathbf{e}_i + \mathbf{B}_1) + \mathbf{B}_2, \\ z_j &= \mathbf{W}_2 \mathrm{Relu}(\mathbf{W}_1 \mathbf{e}_j + \mathbf{B}_1) + \mathbf{B}_2, \end{aligned} \tag{S17}$$

where $\mathbf{W}_1$ and $\mathbf{B}_1$ are the weight and bias of the input layer, respectively. $\mathbf{W}_2$ and $\mathbf{B}_2$ are similarly defined for the hidden layer. $\mathbf{e}_i$ and $\mathbf{e}_j$ denote the input vector of input edges i and j. $\mathbf{O}$ corresponds to the output of CPNN, which is represented as $\mathbf{O} = [o_i, o_j]$, where o_i and o_j represent the probability that i and j are generated later, respectively. Naturally we have $o_i + o_j = 1$.

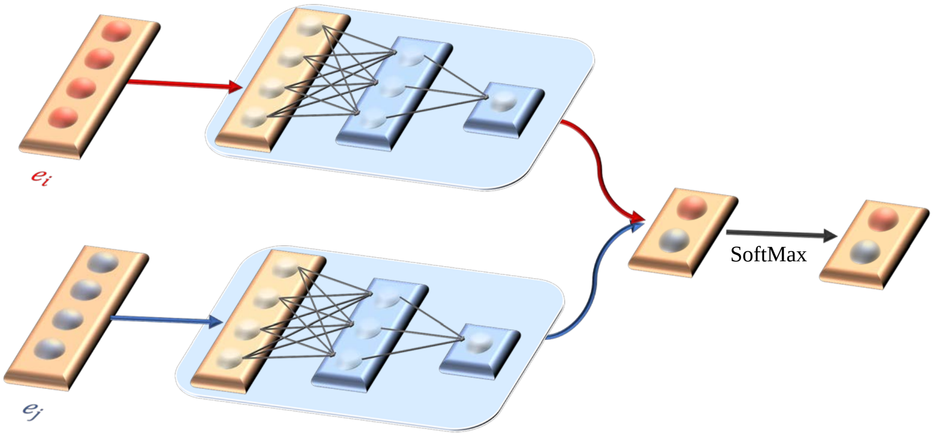

Figure S2: **Architecture of CPNN Model.** The vectors of two given edges i and j propagate forward independently through a fully connected neural network, and output 1-dimensional values respectively. The two outputs are spliced into a 2-dimensional vector and processed by SoftMax to get the final output.

1.2.2 Loss Function of CPNN

In machine learning, the loss function (also known as the cost function) is used to evaluate how well the predicted value is consistent with the ground truth. The smaller the loss function is, the better the algorithm models the dataset. The process of training a neural network is also the process of minimizing the loss function via parameter optimization. In this work, we adopt the cross entropy [19] as the loss function of the CPNN model, which is widely used in classification problems.

In the CPNN model, the loss function consists of two terms. The first one is the value of cross-entropy calculated from the output value of CPNN $\mathbf{O} = [o_i, o_j]$ and the label of the training data $\mathbf{Y} = [y_i, y_j]$:

$$\mathbb{H}(\mathbf{Y}, \mathbf{O}) = -(y_i \log(o_i) + y_j \log(o_j)). \tag{S18}$$

The second part is the regularization term, which is introduced to reduce over-fitting in the training process. We carry out L_2 regularization for the weight and bias of each layer of CPNN, which is formalized as:

$$||\mathbf{W}||_2 = \sqrt{\sum_{l=1}^{n} {w_l}^2}. \tag{S19}$$

where $\mathbf{W} = [w_1, w_2, ..., w_n]$ is the weight vector of a given layer. Overall, the loss function can be represented as:

$$\mathcal{L} = -(y_i \log(o_i) + y_j \log(o_j)) + \eta \sum_{l=1}^{2}(||\mathbf{W}_l||_2^2 + ||\mathbf{B}_l||_2^2)), \tag{S20}$$

where $\mathbf{W}_l$, $\mathbf{B}_l$ are the weight and bias of the l-th layer, respectively. η is the regularization coefficient which is set to 0.001 in this work.

1.2.3 Training process of CPNN

For each training sample, the inputs are vectors of two given edges i and j, while the output is a two-dimension one-hot vector, i.e., [1, 0] if i is generated later and [0, 1] otherwise. We use mini-batch gradient descent to train the CPNN. Specifically, in one epoch, we create a mini-batch of fixed size for the training data. In each iteration, we pick one mini-batch and feed it to CPNN to update the weights. The size of the mini-batch is set to 1% of the total training data, and the minimum size is set to 16. The number of epochs is set to 800. To minimize the loss function, an Adam optimizer [20] is employed in the gradient descent process. The hyper-parameters of the Adam optimizer are set as follows: learning rate=0.001, $\beta_1 = 0.9$, $\beta_2 = 0.999$, $\epsilon = 10^{-8}$.

1.3 Ensemble model

As described in Sec. 1.1, we have six different CPNN base models and one "Best feature" to predict the generation order for edge pairs. Therefore, for each pair of edges, we have seven outputs $\mathbf{O}$. Then, we use an ensemble model to integrate all the results through linear weighting, where the optimal weights are learned and the sum of weights is set to 1. The final prediction result is given by the ensemble model.

Specifically, we take each output o_i^l (i.e., the probability that edge i is generated later) of l-th result as the input of ensemble model, denoted as o_i^1, o_i^2, ..., o_i^7. Then, the output of the ensemble model o_i^{final} is represented as:

$$o_i^{\text{final}} = \sum_{l=1}^{7} w_l o_i^l, \tag{S21}$$

where w_l is the weight of the l-th base result. In the training process, for each possible weight combination, we calculate the accuracy of the corresponding ensemble model on the training set. Then we take the weight combination giving the highest accuracy (i.e., the optimal linear combination) as the weight parameter in the final prediction. The value of each weight takes from 0∼1 at 0.01 intervals.

1.4 Construction of Training set and Test set

In the entire machine learning process, we need to divide the edge pairs that can distinguish the generation order into three sets, including two training sets and one test set. One training set is used to train CPNN base models, while the other one is used to learn the parameters of the ensemble model. These three sets are independent of each other, that is, the generation order between any pair of edges within one set should not be inferred from another set. For example, if edge i is generated later than edge j, edge j is generated later than edge r, and edge pairs (i, j) and (j, r) are in the training set for CPNN model. Then edge pair (i, r) can not be in the training set for the ensemble model because the generation order of i and r is determined by the edge pairs in the CPNN training set.

To construct three independent sets, as shown in Fig. S3, we first split the edges with known time information into three disjoint sets, $\mathbb{E}_1$, $\mathbb{E}_2$ and $\mathbb{E}_3$. Then three edge pair sets $\mathbb{S}_1$, $\mathbb{S}_2$ and $\mathbb{S}_3$ are produced based on the above three edge sets. The specific rules are as follows.

1. $\mathbb{S}_1$ is used for CPNN base model training, which is the set of edge pairs obtained by pairwise combinations of edge set $\mathbb{E}_1$.

2. $\mathbb{S}_2$ is used for ensemble model training, which consists of two parts. One is edge pairs obtained by pairwise combinations of edge set $\mathbb{E}_2$. The other one is edge pairs where one edge is taken from $\mathbb{E}_1$ while the other is taken from $\mathbb{E}_2$.

3. $\mathbb{S}_3$ is used for model testing, which consists of three parts. Edge pairs obtained by pairwise combination of edge set $\mathbb{E}_3$, edge pairs where one link is taken from $\mathbb{E}_1$ while the other is taken from $\mathbb{E}_3$, and edge pairs where one link is taken from $\mathbb{E}_2$ while the other is taken from $\mathbb{E}_3$.

Since the ensemble model has fewer parameters than the CPNN model, the latter requires a larger training set. Therefore, the ratio between $\mathbb{E}_1$ and $\mathbb{E}_2$ is set to 7:1, $\mathbb{E}_1$ and $\mathbb{E}_2$ together account for 40% of the total number of edges in the network.

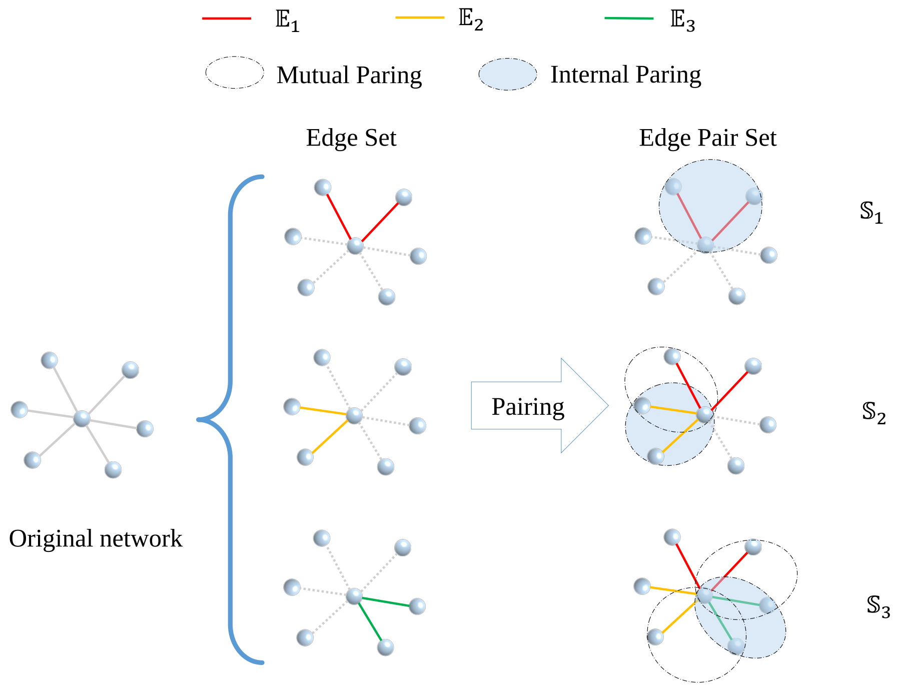

Figure S3: **Construction of training set and test set.** First split edges into three sets $\mathbb{E}_1$, $\mathbb{E}_2$ and $\mathbb{E}_3$. Then construct three edge pair sets: CPNN training set $\mathbb{S}_1$, ensemble model training set $\mathbb{S}_2$ and test set $\mathbb{S}_3$ based on the rules described in Sec. 1.4 such that the three edge pair sets are independent of each other.

2 Ranking methods

After we obtain the generation order of each pair of edges through the machine learning model, a ranking algorithm is needed to infer the generation sequence of all edges based on the edge pair orders. Here we consider a voting-based ranking method – Borda count [21], which aims at aggregating different ranking results into a consistent result. An intuitive example is to determine a movie's rank of the general public. One could design a questionnaire to ask respondents to list the ranking of movies in their minds. However, the ranking of movies, the length of the ranking list, and even the evaluation criteria vary from person to person. How to integrate these lists to get a comprehensive and consistent ranking result is the problem to be solved by Borda count. For each ranking list, score each candidate (i.e., movie) according to its position in the list. The higher the ranking, the higher the score. Then the scores of each candidate in different ranking lists are added up to get a final result, which is called "Borda" number. The final ranking result can be obtained by sorting the Borda numbers of all candidates in descending order.

In our cases, the generation order of each pair of edges can be regarded as a ranking result of two candidates. For each ranking result, the edge that generated later gets one point, and the edge that generated earlier gets zero points. In this way, the score of edge i is:

$$u_i = \sum_{j=1, j \neq i}^{E} M_{ij}. \tag{S22}$$

where E is the number of edges, M_{ij} represents the generation order of edges i and j predicted by the machine learning algorithm. If the prediction result suggests that edge i is added after j, then $M_{ij} = 1$, otherwise $M_{ij} = 0$. After identifying the score of all edges, the edge sequence generated from early to late can be obtained by sorting the edges according to the score in ascending order.

3 Data Set

We list the 17 real-world networks considered in this work and their basic information in Table S1. The networks can be classified into the following five categories: protein-protein interaction (PPI) network, world trade web, collaboration network, animal network, and transportation network. All the networks are undirected and unweighted growing networks. Multiple snapshots of each network are recorded in the dataset. Next, we introduce the details of each network by different network categories.

3.1 Protein-protein interaction (PPI) networks

Five protein-protein interaction (PPI) networks including Fungi (Saccharomyces cerevisiae), Human (Homo sapiens), Fruit fly (Drosophila melanogaster), Worm (Caenorhabditis elegans), and Bacteria (Escherichia coli K-12) are considered in this work [22,27,28]. In each network, a node represents a protein, and an edge between two nodes indicates that there is interaction between the two proteins. A network records the protein-protein interaction relationship of a biological cell in different historical periods. Therefore, we can access edge structures for multiple snapshots of each network. The number of snapshots for a network is shown in Table S1. Note that the time when an edge first appears in the network is recorded as the generation time of the edge. Two edges at different snapshots are the edge pair that can distinguish the generation order. The ratio of the number of generation-order-distinguishable edge pairs E_d to all edge pairs of the network is denoted as P_{E_d}.

The network information we use in this work includes the final topological structure of a network and the generation time of each edge present in the final structure. In the original data of PPI networks, due to the process of protein integration (multiple nodes evolve into one node) and differentiation (one node evolves into multiple nodes), the nodes in the network at different snapshots cannot always keep one-to-one correspondence and thus the exact generation time for some edges are difficult to determine. To solve this problem, we take the network structure at the final snapshot as the benchmark, and convert the nodes that had been merged or diverged at the previous snapshots to align with the final snapshot. For example, if node A appearing at time T_n is merged by node B and C at the previous

Table S1: **Basic information of real-world networks.** From left to right, we report: the network category, network name, number of nodes N, number of edges at final snapshot E, the number of edge pairs that can distinguish the generation order E_d, the ratio of the order-distinguishable edge pairs to the total number of edge pairs $P_{E_d} = E_d/(E(E-1)/2)$, the number of snapshots in a network S, and reference of the dataset.

Network type	Network name	N	E	E_d	P_{E_d}	S	Refs
Protein-Protein Interaction (PPI)	Fungi	2,144	6,000	3,132,599	0.174	3	[22]
	Human	1,891	2,840	1,467,231	0.364	3	
	Fruit Fly	461	598	58,768	0.329	3	
	Worm	485	438	57,980	0.606	4	
	Bacteria	873	2,321	357,730	0.133	2	
World Trade Web	WTW	187	3,249	1,799,895	0.341	17	[23]
Collaboration	Complex Networks	225	413	84,445	0.993	172	[24]
	Chaos	2,055	3,758	7,048,311	0.998	1,118	
	Fluctuations	1,248	2,198	2,408,204	0.997	731	
	Interfaces	2,745	6,718	22,506,659	0.998	1,040	
	Phase Transitions	1,113	1,882	1,764,785	0.997	654	
	Thermodynamics	158	228	25,668	0.992	131	
Animal	Weaver	445	1,332	762,058	0.860	8	[25]
	Ants	102	5,091	824,897	0.064	6	
Transportation	Airplane	48	125	1,291	0.167	5	[26]
	Ferry	216	185	3,427	0.201	6	
	Coach	1,874	2,666	84,495	0.024	4	

time T_{n-1}, we directly convert node B and C at and before time T_{n-1} to node A, and the edges connecting to B or C are all considered as the connections to node A. Similarly, if two nodes B and C at time T_n are differentiated from the node A at the previous time T_{n-1}, we convert node A at and before T_{n-1} to two nodes B and C, and all the edges of A are considered edges connecting to B and C. After aligning nodes at different snapshots to the final snapshot, the generation time of each edge can be determined as the time when the edge first appears.

3.2 World trade web

World Trade Web (WTW) [23] contains the bilateral trade data for each year from 1997 to 2013. Nodes represent countries and edges represent bilateral trade flow between two countries. The time when two countries first report trade between each other is the generation time of the edge between them. In this work, we only care about the generation time of the edges that exist at the final structure of the network, and do not consider the edges that are disconnected during the evolving process.

3.3 Collaboration networks

We use the publication data from the American Physical Society to construct the collaboration networks. According to the Physics and Astronomy Classification Scheme (PACS), published papers can be classified into different fields. We extract data from six different fields, including Complex Networks (PACS = 89.75, 2001 - 2010), Chaos (PACS = 05.45, 1985-2010), Fluctuation (PACS = 05.40, 1980-2010), Interfaces (PACS = 68.35, 1986-2010), Phase Transitions (PACS = 64.60, 1978-2010), and Thermodynamics (PACS = 05.70, 1978-2010) [24]. The networks are then constructed by considering the cooperation relationship between authors. The nodes in the network represent the authors of the article. If two authors are coauthors of two or more papers, they will be connected by an edge. We denote the generation time of the edge as the date of the earliest collaborative publication between the two authors.

3.4 Animal networks

Two animal networks are used in this work, they are weaver (philetairus socius) interaction network [25, 29] and ants (camponotus fellah) interaction network [25, 30].

The weaver interaction network contains 10 months of social data collected from 23 colonies of weavers in South Africa, in which nodes represent weavers and edges added between two nodes indicate the two weavers had interaction with each other, i.e., they used the same nest chamber. The generation time of the edge is defined as the timestamp of the earliest interaction recorded between two weavers. There are a total of 23 snapshots in the original network data. Since there are too few newly added edges at some snapshots, we merge every three consecutive snapshots in the original data together into one snapshot. The final network we get has eight snapshots, i.e. $S = 8$.

The ants interaction network collects interaction (physical contact) data of ants from six colonies of ants during 41 days. Each node represents an ant and each edge represents the interaction between two ants. The position and orientation of all ants are recorded twice per second to infer the social interactions of ants. The original data contains 41 snapshots. Similar to the weaver network, too few new edges are generated at one snapshot. To reduce the sparsity of the original data, we combine the data of every three time points into one. The final network has 14 snapshots. Since some snapshots have no new edges added, the number of effective snapshots is only six. The generation time of each edge in the network is defined as its earliest appearance time in the network.

3.5 Transportation networks

A networked system of transportation is obtained from the Reference [26] which records traffic data in the UK for a week in October 2010. The original data is represented as a multiple-layer network, each of which corresponds to a specific vehicle, such as an airplane, bus, private car, etc. In this work, we extract three networks from the original data, including airplane, ferry, and coach transportation networks. The nodes in the networks represent airports, ferry docks, and coach stations, respectively. The edges indicate that there is at least one flying, sailing, or transporting event that happened between two nodes in the corresponding network. The earliest time of the transportation event between two nodes is recorded as the generation time of the edge. The original data is directed, here we ignore the directions for simplicity. The time in the original data is recorded per minute, which leads to too few edges at each time point. Therefore, we merge all the time points within one day as one time point, resulting in 7 snapshots for each network. Considering there are no new edges added at some snapshots, the number of effective snapshots is five for the airplane network, six for the ferry network, and four for the coach network.

4 Edge Restoration results on real-world networks.

In this section, we show the edge restoration results of different real-world networks by our method.

First, we visualize the generation order of the edges restored using our approach for some real networks, including the collaboration network (Complex networks, CN, see Fig. S4), PPI network for fungi (Fig. S5)) and PPI network for worm (Fig. S6). The big gap between the restored and the random sequences along with the high concordance between the restored and the real sequences in Fig. S4 demonstrate that our model is able to learn useful information from partial history and recover the full network formation history quite well. For PPI networks with fungi and worm, the real data contain only a few snapshots, so visualizations of the real sequences are not informative due to their coarse granularity so that we plot how the average degree of nodes changes as E increases (see Fig. S7). It is found that the real snapshots fall closely onto the curve of the restored outcome using our model, again indicating that our model is capable of recovering the real situation well.

Figure S7 shows the average degree of nodes as the increase of edges for all real-world networks. It can be seen that at the beginning of the network growth, the edges are usually added to a few nodes in the real situation. And our restored edge sequences can demonstrate this mechanism very well (the yellow triangles and blue lines fit well for most networks).

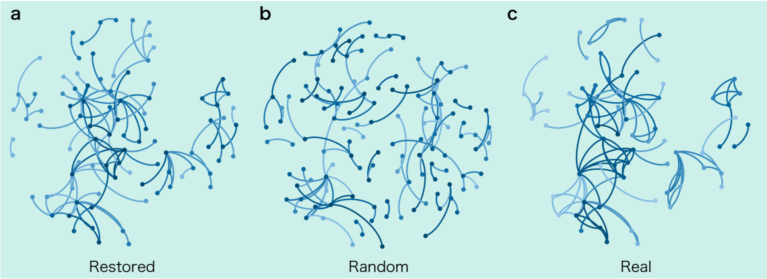

Figure S4: **Restored formation processes of collaboration network in the field of complex network (CN).** Visualizations of the first 100 edges in the collaboration network (CN) based on **a** the restored generation process, **b** a random sequence, and **c** the ground truth, respectively. The darker edge color represents that the edge is added earlier to the network.

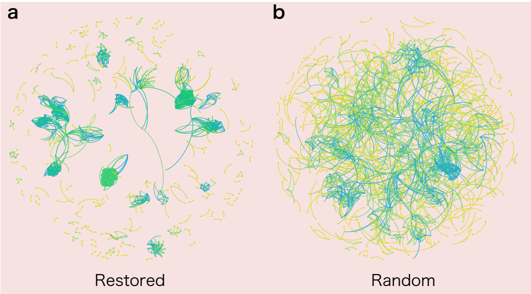

Figure S5: **Restored formation processes of PPI network for fungi.** Visualizations of the first 1500 edges in the PPI network for fungi based on **a** the restored generation process and **b** a random sequence. The darker edge color represents that the edge was added earlier to the network.

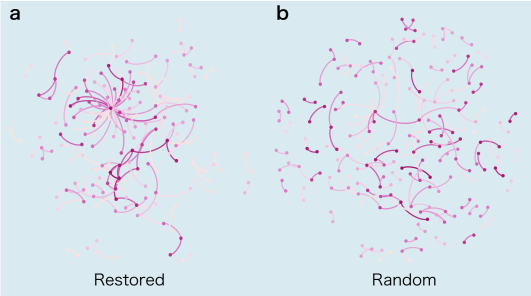

Figure S6: **Restored formation processes of PPI network for worm.** Visualizations of the first 150 edges in the PPI network for worm based on **a** the restored generation process and **b** a random sequence. The darker edge color represents that the edge was added earlier to the network.

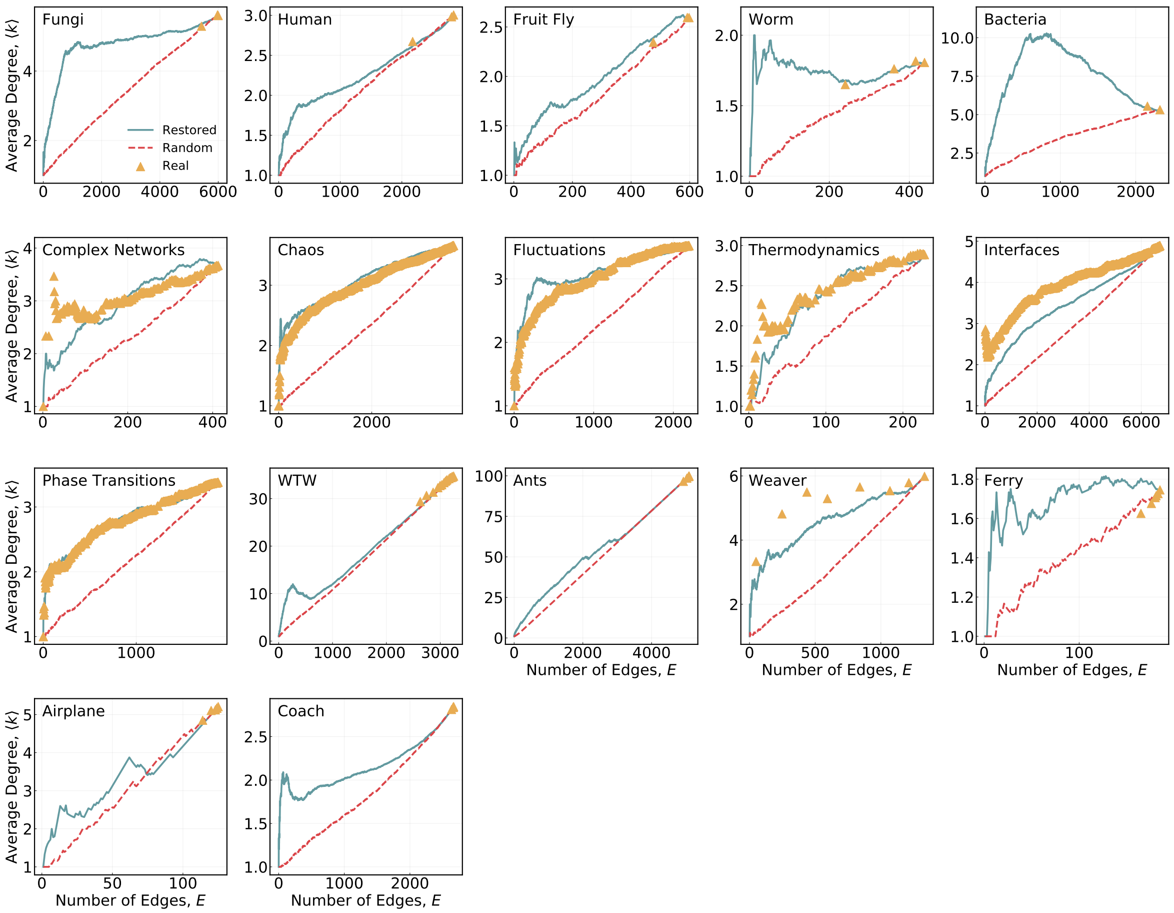

Figure S7: **Average degree of nodes for real-world networks.** The yellow triangles, blue solid lines, and red dashed lines are results based on real edge generation order, edge generation order produced by our method and by random assignment, respectively.

5 Accuracy of the machine learning algorithm to predict generation order of edge pairs

5.1 Accuracy of different methods on different real-world networks

In this section, we show the accuracy of each single CPNN base model, best feature, and ensemble model to predict the generation order of edge pairs on different real-world networks. For each network and each method, the results are averaged by 100 simulations. As shown in Table S2, in most networks,

the ensemble model is the method with the highest accuracy.

Table S2: **Accuracy of different methods on real-world networks.** From left to right, we report the network name, the accuracy of the best feature, the CPNN base model with collection feature, DeepWalk, Node2Vec, LINE, Struc2Vec, SDNE, and the ensemble model. Each result is averaged by 100 simulations. The standard deviation is also shown in the table. For each network, the highest result is highlighted in boldface.

Network name	Best Feature	Collection Feature	DeepWalk	Node2Vec	LINE	Struct2Vec	SDNE	Ensemble
Fungi	0.650 ± 0.003	0.699 ± 0.011	0.856 ± 0.005	0.851 ± 0.008	0.691 ± 0.012	0.756 ± 0.009	0.819 ± 0.007	$\mathbf{0.877 \pm 0.007}$
Human	0.591 ± 0.009	0.698 ± 0.006	0.878 ± 0.008	0.885 ± 0.007	0.767 ± 0.010	0.738 ± 0.015	0.819 ± 0.011	$\mathbf{0.897 \pm 0.008}$
Fruit Fly	0.622 ± 0.007	0.635 ± 0.030	0.790 ± 0.021	$\mathbf{0.801 \pm 0.018}$	0.771 ± 0.028	0.712 ± 0.017	0.714 ± 0.022	0.797 ± 0.029
Worm	0.545 ± 0.013	0.610 ± 0.012	0.697 ± 0.022	0.700 ± 0.029	0.693 ± 0.015	0.694 ± 0.028	0.707 ± 0.018	$\mathbf{0.742 \pm 0.025}$
Bacteria	0.770 ± 0.005	0.821 ± 0.015	0.878 ± 0.022	0.878 ± 0.024	0.781 ± 0.012	0.846 ± 0.011	0.878 ± 0.007	$\mathbf{0.909 \pm 0.010}$
WTW	0.821 ± 0.002	0.873 ± 0.002	0.839 ± 0.007	0.552 ± 0.034	0.768 ± 0.007	0.902 ± 0.004	0.640 ± 0.007	$\mathbf{0.910 \pm 0.007}$
Complex Networks	0.549 ± 0.010	0.582 ± 0.013	$\mathbf{0.749 \pm 0.018}$	0.737 ± 0.020	0.671 ± 0.020	0.654 ± 0.016	0.692 ± 0.013	0.745 ± 0.023
Chaos	0.547 ± 0.001	0.573 ± 0.002	0.793 ± 0.003	0.775 ± 0.003	0.644 ± 0.006	0.663 ± 0.004	0.684 ± 0.004	$\mathbf{0.795 \pm 0.003}$
Fluctuations	0.538 ± 0.002	0.565 ± 0.005	0.849 ± 0.003	0.824 ± 0.003	0.667 ± 0.002	0.722 ± 0.006	0.719 ± 0.004	$\mathbf{0.852 \pm 0.002}$
Interfaces	0.559 ± 0.002	0.595 ± 0.003	0.857 ± 0.008	0.830 ± 0.004	0.645 ± 0.010	0.702 ± 0.007	0.747 ± 0.005	$\mathbf{0.858 \pm 0.008}$
Phase Transitions	0.570 ± 0.003	0.599 ± 0.004	0.820 ± 0.022	0.808 ± 0.018	0.681 ± 0.023	0.694 ± 0.027	0.723 ± 0.014	$\mathbf{0.821 \pm 0.020}$
Thermodynamics	0.604 ± 0.020	0.666 ± 0.016	$\mathbf{0.846 \pm 0.008}$	0.837 ± 0.010	0.776 ± 0.018	0.710 ± 0.026	0.739 ± 0.013	0.838 ± 0.012
Weaver	0.566 ± 0.006	0.665 ± 0.013	0.984 ± 0.003	0.982 ± 0.004	0.816 ± 0.008	0.824 ± 0.012	0.905 ± 0.005	$\mathbf{0.985 \pm 0.004}$
Ants	0.751 ± 0.004	0.763 ± 0.005	0.687 ± 0.011	0.517 ± 0.018	0.737 ± 0.022	0.786 ± 0.013	0.813 ± 0.017	$\mathbf{0.841 \pm 0.014}$
Airplane	0.830 ± 0.042	$\mathbf{0.917 \pm 0.022}$	0.883 ± 0.037	0.914 ± 0.027	0.855 ± 0.062	0.871 ± 0.032	0.869 ± 0.039	0.882 ± 0.041
Ferry	0.620 ± 0.019	0.680 ± 0.065	0.789 ± 0.039	$\mathbf{0.824 \pm 0.044}$	0.691 ± 0.045	0.734 ± 0.046	0.800 ± 0.028	0.795 ± 0.028
Coach	0.808 ± 0.009	0.765 ± 0.048	0.898 ± 0.022	$\mathbf{0.921 \pm 0.020}$	0.772 ± 0.036	0.694 ± 0.068	0.750 ± 0.063	0.877 ± 0.043

5.2 Influence of training ratio on the prediction accuracy

We test the influence of the training ratio ω on the accuracy for different CPNN base models, the best feature, and the ensemble model. The results on real-world networks are shown in Fig. S8. Note that for different networks, the proportion of edge pairs with distinguishable generation order to all edge pairs is different, so that their ranges of the x-axis are also different.

5.3 Accuracy of CPNN model with single edge index

In this study, we combine the 11 edge indexes into an 11-dimensional vector (collection feature) as the input of the CPNN machine learning model to predict the generation order of edge pairs. For comparison, we also train the CPNN model with each single edge index (single feature) and show its accuracy in Tab. S3. It can be seen that the accuracy of collection features is higher than single features in general.

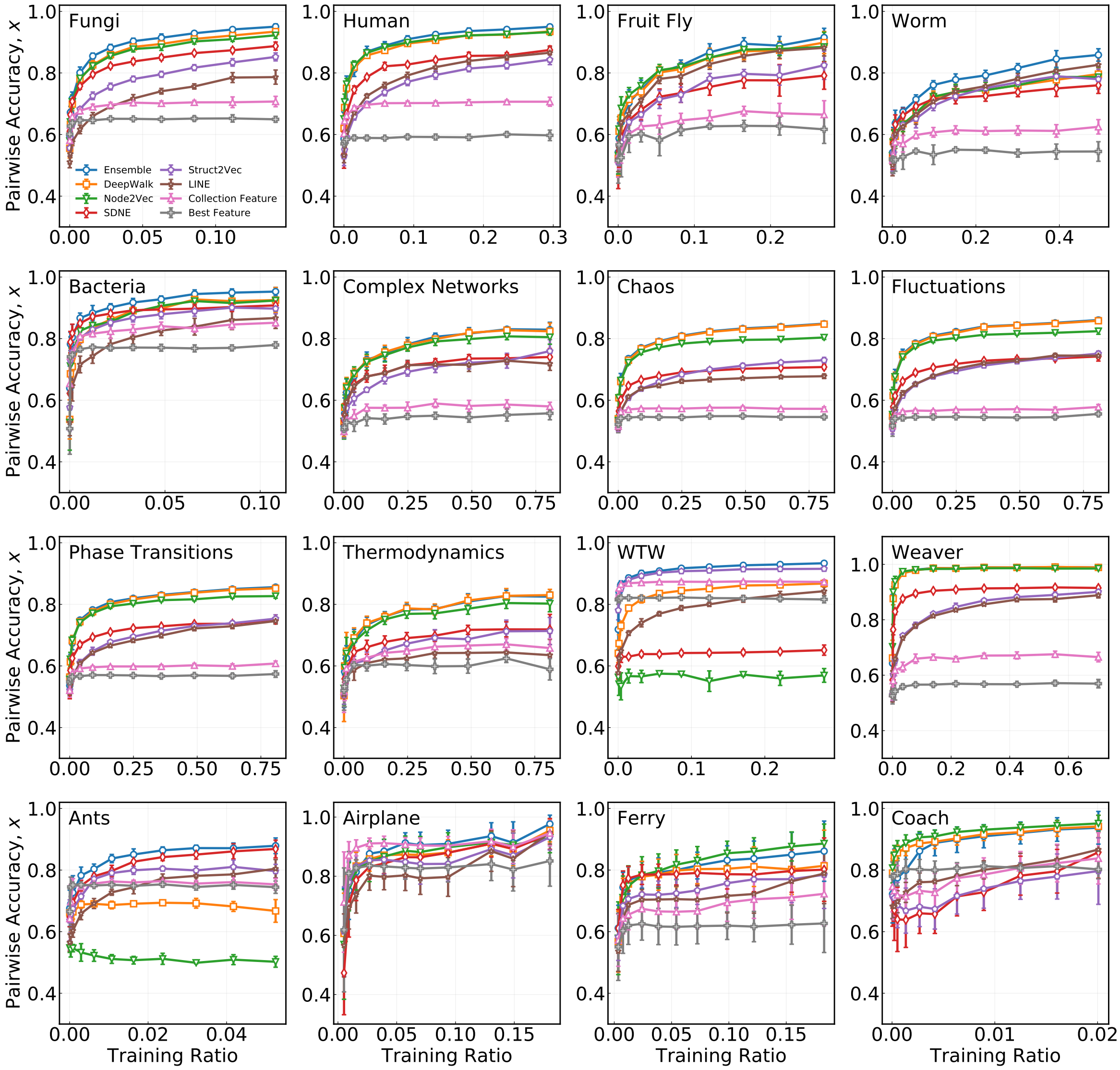

Figure S8: **The influence of training ratio on the prediction accuracy for different methods.** The accuracy of different methods as a function of the training ratio on real-world networks. The points are averaged by 100 simulations, and the error bar shows the standard deviation.

Table S3: **Accuracy of CPNN model with single edge index.** From left to right, we report network name, accuracy of edge betweenness (BN), edge degree (DEG), common neighbor (CN), edge clustering coefficient (CC), edge strength (STR), resource allocation index (RA), Adamic-Adar index (AA), preferential attachment index (PA), local path index (LP), edge PageRank (PR), edge k-shell (KS) and collection feature (Collection). Each result is averaged by 100 simulations. The standard deviation is also shown in the table. For each network, the highest result is highlighted in boldface. Note that the accuracy shown in this paper is obtained on the test set unless otherwise stated.

Network name	BN	DEG	CN	CC	STR	RA	AA	PA	LP	PR	KS	Collection
Fungi	0.62 ± 0.00	0.61 ± 0.01	0.62 ± 0.06	0.62 ± 0.01	0.61 ± 0.06	0.63 ± 0.04	0.64 ± 0.04	0.60 ± 0.03	0.60 ± 0.07	0.53 ± 0.01	0.62 ± 0.00	$\mathbf{0.70 \pm 0.01}$
Human	0.58 ± 0.04	0.56 ± 0.03	0.55 ± 0.00	0.56 ± 0.03	0.57 ± 0.02	0.56 ± 0.02	0.56 ± 0.02	0.56 ± 0.03	0.52 ± 0.02	0.52 ± 0.08	0.51 ± 0.02	$\mathbf{0.70 \pm 0.01}$
Fruit Fly	0.53 ± 0.05	0.60 ± 0.05	0.52 ± 0.02	0.50 ± 0.01	0.49 ± 0.01	0.50 ± 0.01	0.49 ± 0.02	0.57 ± 0.05	0.54 ± 0.03	0.58 ± 0.06	0.51 ± 0.01	$\mathbf{0.64 \pm 0.03}$
Worm	0.53 ± 0.03	0.55 ± 0.02	0.51 ± 0.01	0.51 ± 0.01	0.51 ± 0.01	0.51 ± 0.01	0.51 ± 0.01	0.54 ± 0.03	0.51 ± 0.03	0.54 ± 0.02	0.52 ± 0.01	$\mathbf{0.61 \pm 0.01}$
Bacteria	0.59 ± 0.03	0.59 ± 0.04	0.73 ± 0.08	0.69 ± 0.06	0.69 ± 0.10	0.74 ± 0.01	0.70 ± 0.10	0.70 ± 0.07	0.76 ± 0.01	0.49 ± 0.01	0.75 ± 0.08	$\mathbf{0.82 \pm 0.01}$
WTW	0.64 ± 0.07	0.76 ± 0.00	0.71 ± 0.14	0.54 ± 0.02	0.67 ± 0.11	0.74 ± 0.12	0.77 ± 0.09	0.79 ± 0.10	0.81 ± 0.00	0.65 ± 0.00	0.73 ± 0.12	$\mathbf{0.87 \pm 0.00}$
Complex Networks	0.50 ± 0.01	0.50 ± 0.01	0.52 ± 0.01	0.51 ± 0.01	0.52 ± 0.01	0.55 ± 0.02	0.54 ± 0.02	0.51 ± 0.01	0.51 ± 0.01	0.50 ± 0.00	0.51 ± 0.01	$\mathbf{0.58 \pm 0.01}$
Chaos	0.55 ± 0.00	0.52 ± 0.01	0.51 ± 0.01	0.53 ± 0.01	0.53 ± 0.02	0.50 ± 0.01	0.49 ± 0.01	0.52 ± 0.00	0.49 ± 0.01	0.53 ± 0.01	0.52 ± 0.01	$\mathbf{0.57 \pm 0.00}$
Fluctuations	0.54 ± 0.01	0.53 ± 0.01	0.51 ± 0.01	0.55 ± 0.00	0.53 ± 0.01	0.51 ± 0.01	0.51 ± 0.01	0.53 ± 0.01	0.50 ± 0.00	0.54 ± 0.00	0.51 ± 0.00	$\mathbf{0.56 \pm 0.01}$
Interfaces	0.54 ± 0.00	0.52 ± 0.01	0.54 ± 0.02	0.53 ± 0.00	0.55 ± 0.00	0.53 ± 0.01	0.55 ± 0.01	0.52 ± 0.01	0.55 ± 0.02	0.51 ± 0.01	0.54 ± 0.03	$\mathbf{0.59 \pm 0.00}$
Phase Transitions	0.56 ± 0.03	0.51 ± 0.01	0.54 ± 0.02	0.55 ± 0.02	0.57 ± 0.00	0.54 ± 0.02	0.55 ± 0.00	0.50 ± 0.00	0.54 ± 0.01	0.55 ± 0.01	0.54 ± 0.02	$\mathbf{0.60 \pm 0.00}$
Thermodynamics	0.56 ± 0.02	0.58 ± 0.05	0.51 ± 0.02	0.55 ± 0.04	0.54 ± 0.05	0.51 ± 0.03	0.50 ± 0.03	0.58 ± 0.01	0.50 ± 0.01	0.57 ± 0.04	0.50 ± 0.02	$\mathbf{0.67 \pm 0.02}$
Weaver	0.55 ± 0.02	0.56 ± 0.02	0.54 ± 0.01	0.50 ± 0.01	0.50 ± 0.01	0.50 ± 0.01	0.51 ± 0.01	0.56 ± 0.00	0.54 ± 0.02	0.53 ± 0.01	0.52 ± 0.02	$\mathbf{0.67 \pm 0.01}$
Ants	0.73 ± 0.08	0.72 ± 0.07	0.72 ± 0.07	0.59 ± 0.00	0.73 ± 0.08	0.74 ± 0.01	0.74 ± 0.01	0.62 ± 0.12	0.72 ± 0.07	0.56 ± 0.02	0.63 ± 0.04	$\mathbf{0.76 \pm 0.01}$
Airplane	0.68 ± 0.15	0.83 ± 0.11	0.53 ± 0.03	0.76 ± 0.09	0.69 ± 0.12	0.49 ± 0.03	0.51 ± 0.04	0.73 ± 0.15	0.51 ± 0.04	0.82 ± 0.11	0.63 ± 0.08	$\mathbf{0.92 \pm 0.02}$
Ferry	0.51 ± 0.02	0.58 ± 0.06	0.62 ± 0.01	0.62 ± 0.01	0.60 ± 0.05	0.60 ± 0.04	0.60 ± 0.03	0.56 ± 0.09	0.61 ± 0.07	0.54 ± 0.02	0.57 ± 0.04	$\mathbf{0.68 \pm 0.07}$
Coach	0.54 ± 0.04	0.74 ± 0.14	0.69 ± 0.07	0.68 ± 0.03	0.64 ± 0.08	0.65 ± 0.07	0.67 ± 0.06	$\mathbf{0.78 \pm 0.10}$	0.73 ± 0.12	0.74 ± 0.01	0.76 ± 0.01	0.77 ± 0.05

5.4 Effect of coarse-grained edge generation time on the edge pair prediction accuracy

In the real world, networks usually lack the exact generated time of each edge. The generation time of multiple edges is often recorded at the same timestamp. That is, the generation time of edges in the network is coarse-grained. In order to study the effect of the coarse-grained edge generation time on the ability of our method to infer the edge formation process of a network, we carry out some experiments on BA networks with different degrees of coarse-grained edge generation time. Specifically, as shown in Fig. S9, all edges of a BA network are sorted according to their generation time from early to late and then evenly divided into S bins. Edges falling into the same bin share the same generation time. We train the ensemble model using the coarse-grained BA network and calculate the accuracy of the ensemble model on the test set.

In our experiments, the BA network has 2000 nodes and 19915 edges. The number of bins S is tuned to control the degree of the time coarsening. Here we use time coarsened networks with $S = 3, 5, 7$ and the original network ($S = 1991$) to train the ensemble model and calculate the accuracy on the test set, respectively. As shown in Tab. S4, the accuracy grows as the degree of coarse-grain decreases (S increases). More importantly, even though the edge time of a network is highly coarse-grained, the

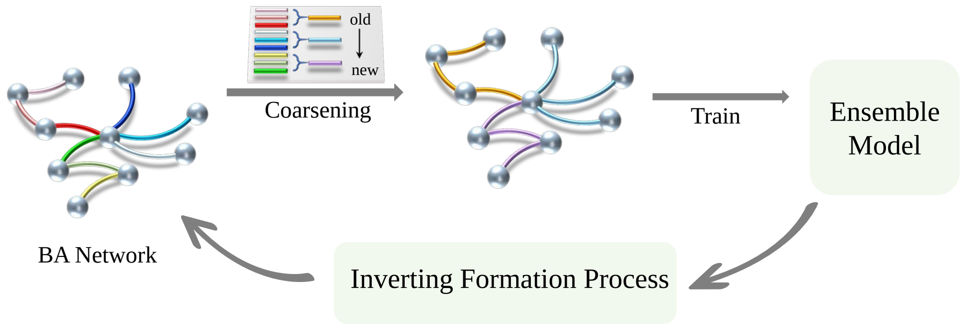

Figure S9: **Illustration of coarsening the edge generation time on BA network** All edges of a BA network are sorted according to their generation time from early to late and then evenly divided into T bins. Train the ensemble model using the time coarse-grained network. Then inverting the edge formation process based on the results of the ensemble model.

accuracy to distinguish the edge generation order is still very high.

Table S4: **Accuracy of the ensemble model trained by the time coarse-grained networks.** Smaller S represents the degree of the coarse-grain is higher. For the original network, $S = 1991$.

BA network	$S = 3$	$S = 5$	$S = 7$	Original ($S = 1991$)
Accuracy	0.8257	0.8280	0.8496	0.8938

6 Overall error of the restored edge sequence

6.1 Theoretical relation between the prediction accuracy of two edges' generation order and the overall error $\mathcal{E}$

In this section, we introduce the relation between the accuracy of two edges' generation order predicted by the ensemble model and the overall error of the restored edge sequence. For a given number of the edges E, suppose the edges are generated in order, we define the normalized generation order sequence $\boldsymbol{\alpha} = (\alpha_1, \alpha_2, ..., \alpha_E) = (\frac{1}{E}, \frac{2}{E}, ..., 1)$, where α_i is the normalized generation order of edge i, larger α_i means that the edge i joined the network later. In addition, the accuracy of the ensemble model is set to be x, that is, the probability of predicting the generation order of each edge pair correctly is x. Based on the prediction of all $\binom{E}{2}$ edge pairs and the voting-based ranking method (Borda count), we can get an estimated edge generation order sequence $\widehat{\boldsymbol{\alpha}} = (\widehat{\alpha}_1, \widehat{\alpha}_2, ..., \widehat{\alpha}_E)$, where $\widehat{\alpha}_i$

represents the normalized generation order of i.

In Borda count, denote u_{ij} as the score of edge i when comparing the order of edge i and j, u_{ij} equals to $1/E$ if i is added to the network later than j, u_{ij} equals to 0 otherwise. In case of partial edge pairs being misjudged (the probability is $1-x$), the mathematical expectation $\mathbf{E}(u_{ij})$ and variance $\mathbf{Var}(u_{ij})$ of u_{ij} can be written as:

$$\begin{cases} \mathbf{E}(u_{ij}) = \frac{x}{E}, \text{if } \alpha_i > \alpha_j \\ \mathbf{E}(u_{ij}) = \frac{1-x}{E}, \text{if } \alpha_i < \alpha_j \end{cases} \tag{S23}$$

$$\mathbf{Var}(u_{ij}) = \frac{x(1-x)}{E^2}. \tag{S24}$$

Then, the total score of edge i is defined as $u_i = \sum_{j=1,j\neq i}^{E} u_{ij}$. According to Eq. (S23), when edge i is compared with the first $i-1$ edges in the real ranking, the expected score of edge i is x/E, while when edge i is compared with the last $E-i$ edge in the real ranking, the expected score of edge i is $(1-x)/E$. Therefore, the expectation of u_i is:

$$\begin{aligned} \mathbf{E}(u_i) &= \sum_{j=1,j\neq i}^{E} \mathbf{E}(u_{ij}) \\ &= (i-1)\frac{x}{E} + (E-i)\frac{1-x}{E} \\ &= \frac{2x-1}{E}i + 1 - \frac{E+1}{E}x \\ &\approx \frac{2x-1}{E}i + 1 - x \end{aligned} \tag{S25}$$

In the above equation, we approximate $(E+1)/E$ to 1 for large E.

Since E and x are constant values, $\mathbf{E}(u_i)$ is a linear function of i with two boundaries $\mathbf{E}(u_1) = 1-x$ and $\mathbf{E}(u_E) = x$. In other words, the scores of E edges are evenly distributed over the interval of $[1-x, x]$, i.e.,

$$P(u) = \frac{1}{2x-1}, 1-x \leq u_i \leq x. \tag{S26}$$

According to Eq. (S26), $P(u)$ can be regarded as a mean field. Then the comparison of scores for different edges can be simplified to the comparison between u_i and the mean field. According to the mean field theory, the position $\widehat{\alpha}_i$ of i in the sequence $\widehat{\boldsymbol{\alpha}}$ is equivalent to the ratio of the length from $1-x$ to u_i to the total length $2x-1$, i.e.,

$$\widehat{\alpha}_i = \frac{u_i - (1-x)}{2x-1}. \tag{S27}$$

Using Eq. (S27), the variance of $\widehat{\alpha}_i$ can be calculated,

$$\begin{aligned} \mathbf{Var}(\widehat{\alpha}_i) &= \frac{\mathbf{Var}(u_i)}{(2x-1)^2} \\ &= \frac{\sum_j \mathbf{Var}(u_{ij})}{(2x-1)^2}. \end{aligned} \tag{S28}$$

Substituting Eq. (S24) into Eq. (S28), we have:

$$\mathbf{Var}(\widehat{\alpha}_i) = \frac{(1-x)x}{E(2x-1)^2}. \tag{S29}$$

Accordingly, the standard derivation of $\widehat{\alpha}_i$ is given by:

$$\mathbf{Std}(\widehat{\alpha}_i) = \frac{\sqrt{x(1-x)}}{2x-1}\frac{1}{\sqrt{E}} \tag{S30}$$

Also, the expectation of $\widehat{\alpha}_i$ can be calculated from Eq. (S27):

$$\mathbf{E}(\widehat{\alpha}_i) = \frac{\mathbf{E}(u_i) - (1-x)}{2x-1} = \frac{i}{E} = \alpha_i \tag{S31}$$

From Eq. (S31), we can see that the position of edge i in the restored sequence $\widehat{\boldsymbol{\alpha}}$ is an unbiased estimate of which in the real sequence. Therefore, the error of $\widehat{\alpha}_i$ relative to α_i should theoretically be equivalent to the standard deviation of the distribution of $\widehat{\alpha}_i$, i.e.,

$$\mathcal{E}^{\text{theory}} = \frac{\sqrt{x(1-x)}}{2x-1}\frac{1}{\sqrt{E}}. \tag{S32}$$

It is worth noting that for a fixed number of edges E, the theoretical overall error in Eq. (S32) diverges when x approaches $\frac{1}{2}$ and there is a value of x below which the error becomes larger than one. Therefore, in the following, we would like to discuss the domain of validity of Eq. (S32), i.e., Eq.(2) in the main text.

Equation (S24) gives the variance of u_{ij} (the score of edge i when comparing the order of edge i and j in Borda count). Then, the variance and standard deviation of the total score of edge i, $u_i = \sum_{j=1,j\neq i}^{E} u_{ij}$, are

$$\mathbf{Var}(u_i) = \sum_j \mathbf{Var}(u_{ij}) = \frac{x(1-x)}{E}, \tag{S33}$$

$$\mathbf{Std}(u_i) = \sqrt{\frac{x(1-x)}{E}}. \tag{S34}$$

It has been shown that the scores of the edges are uniformly distributed over the interval $[1-x, x]$, which is of width $2x-1$. When $2x-1$ is too small, the variation range of u_i (i.e., $\mathbf{Std}(u_i)$) would go beyond the interval, such that our derivation under this scenario is not valid. Therefore, our derivation is only valid under the condition that $\mathbf{Std}(u_i)$ is much smaller than the width of the interval, i.e.,

$$\sqrt{\frac{x(1-x)}{E}} \ll 2x-1. \tag{S35}$$

Let $x = 0.5 + \delta$, then the above inequality is easily satisfied when δ is not too small. So, we only consider the case when δ is very small so that $x \approx 0.5$. In this case $x(1-x) \approx \frac{1}{4}$, and plugging this into Eq. (S35) we have

$$\delta \gg \frac{1}{4\sqrt{E}}. \tag{S36}$$

Eq. (S36) is the domain of validity of Eq. (S32). Figure S10 illustrates the correctness of Eq. (S32) when Eq. (S36) is satisfied: the simulation results are consistent with that from Eq. (S32) when $\delta\sqrt{E} \gg 1/4$; when $\delta\sqrt{E} \ll 1/4$, the overall error of the simulation results is close to random guessing, i.e., $\mathcal{E} = 1/\sqrt{6}$. According to Fig. S10, Equation (S32) fits the simulation results well when $\delta\sqrt{E} > 2$.

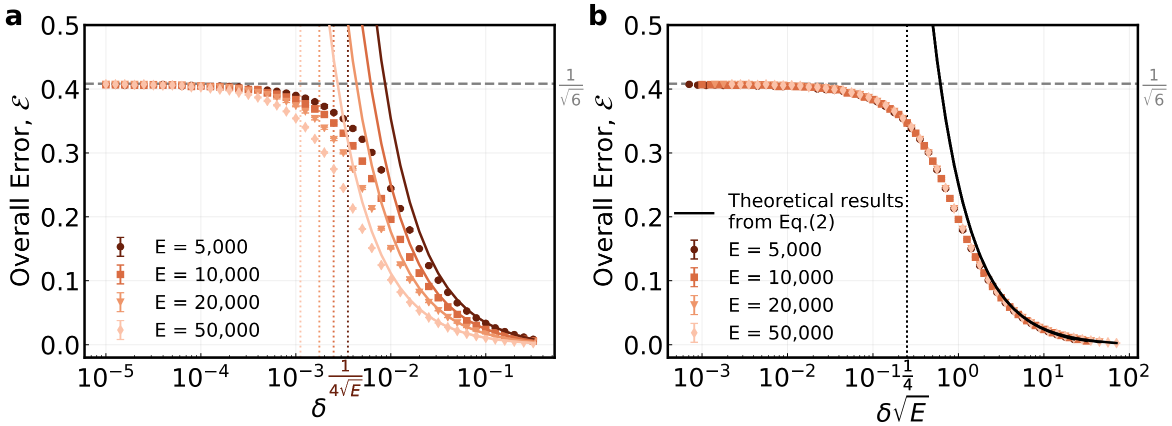

Figure S10: The relationship between the overall error $\mathcal{E}$ and **a** δ, **b** $\delta\sqrt{E}$. In **a**, different colored solid lines are the theoretical results of Eq. (S32) with different values of E and the dotted vertical lines show the corresponding positions of $\delta = 1/4\sqrt{E}$. In **b**, the black solid line is the theoretical results obtained from Eq. (S32) (results with different E overlap) and the dotted vertical line shows the position of $\delta\sqrt{E} = 1/4$. Different colored symbols represent the simulation results with different values of E, where each point represents the average of 10 simulation runs. The error bars represent the standard deviation. The grey dashed line indicates the overall error of random guessing, i.e., $\mathcal{E} = 1/\sqrt{6}$.

Equation (S32) suggests that when the number of edges is large enough, the accuracy of the machine learning model for predicting the relative generation order of any two edges slightly better than random guess is good enough to make the overall error small. Here we provide an intuitive explanation on this statement. Consider edge i and edge j where $\alpha_i > \alpha_j$. In the first step of our method, for any edge l with $\alpha_i > \alpha_l > \alpha_j$, the probability of correctly obtaining the reconstructed pairwise order of both edge pairs (i, l) and (j, l) is x^2, which is the largest among all four possible combinations of pairwise orders since $x > 0.5$ (see Table S5). Therefore, when $\alpha_i - \alpha_j$ is not too small, by the law of large numbers, we could generally obtain sufficient intermediate edges that have correct reconstructed pairwise orders with edge i and edge j, enabling the correct reconstructed ranking $\widehat{\alpha}_i > \widehat{\alpha}_j$ in the vote-based ranking algorithm of the second step. This can be more easily understood through an analogy with a biased dice. Imagine a four-sided dice, one of which has a higher probability than the other three sides. As long as you roll the dice for enough number of times, you are able to detect that the dice is biased.

Table S5: The ranking result of edge i and edge j based on the reconstructed pairwise order of edge pairs (i, l) and (j, l) for any edge l with $\alpha_i > \alpha_l > \alpha_j$.

Reconstructed order of (i, l)	Reconstructed order of (j, l)	Ranking result of edge i and edge j	Probability
$\widehat{\alpha}_i > \widehat{\alpha}_l$	$\widehat{\alpha}_l > \widehat{\alpha}_j$	$\widehat{\alpha}_i > \widehat{\alpha}_j$ (correct)	x^2
$\widehat{\alpha}_i > \widehat{\alpha}_l$	$\widehat{\alpha}_l < \widehat{\alpha}_j$	None	$x(1-x)$
$\widehat{\alpha}_i < \widehat{\alpha}_l$	$\widehat{\alpha}_l > \widehat{\alpha}_j$	None	$x(1-x)$
$\widehat{\alpha}_i < \widehat{\alpha}_l$	$\widehat{\alpha}_l < \widehat{\alpha}_j$	$\widehat{\alpha}_i < \widehat{\alpha}_j$ (incorrect)	$(1-x)^2$

6.2 Equivalence between overall error $\mathcal{E}$ and other correlation coefficients

In this section, we compare our overall error $\mathcal{E}$ with other widely accepted measures for evaluating the correlation between two ordered sequences, including the Kendall's τ [31] and the Spearman's ρ [32]. Theoretically, We find that $\mathcal{E}$ has a monotonic functional relationship with the other measures, suggesting that they are essentially equivalent but differ in value.

6.2.1 Theoretical equivalence between $\mathcal{E}$ and Kendall's τ.

By definition, Kendall's τ between two ordered sequences, e.g., $\boldsymbol{\alpha}$ and $\widehat{\boldsymbol{\alpha}}$, is

$$\tau = 1 - \frac{2(\text{number of discordant pairs})}{\text{number of pairs}}. \tag{S37}$$

The number of pairs is $\binom{E}{2} = E(E-1)/2$, denote the number of discordant pairs by K_d, then

$$K_d = |\{(i, j) : i < j, (\alpha_i < \alpha_j \wedge \widehat{\alpha}_i > \widehat{\alpha}_j) \vee (\alpha_i > \alpha_j \wedge \widehat{\alpha}_i < \widehat{\alpha}_j)\}|. \tag{S38}$$

We have shown in Sec. 6.1 that the expectation and variance of $\widehat{\alpha}_i$ are

$$\mathbf{E}(\widehat{\alpha}_i) = \alpha_i, \mathbf{Var}(\widehat{\alpha}_i) = \frac{(1-x)x}{E(2x-1)^2}. \tag{S39}$$

So, $\widehat{\alpha}_i$ fluctuates around α_i and the range of fluctuation is proportional to

$$\sigma = \sqrt{\mathbf{Var}(\widehat{\alpha}_i)} = \frac{\sqrt{(1-x)x}}{(2x-1)}\sqrt{\frac{1}{E}}. \tag{S40}$$

Similarly, $\widehat{\alpha}_j$ fluctuates around α_j and the range of fluctuation is proportional to σ. Therefore, the proportion of discordant pairs where $(\widehat{\alpha}_i, \widehat{\alpha}_j)$ demonstrates a different order with (α_i, α_j), is proportional to σ:

$$\frac{K_d}{E(E-1)/2} \propto \sigma = \frac{\sqrt{(1-x)x}}{(2x-1)}\sqrt{\frac{1}{E}} \propto \mathcal{E}^{\text{theory}}. \tag{S41}$$

Therefore, we have that

$$\tau = 1 - \frac{2K_d}{E(E-1)/2} = 1 - b\mathcal{E}^{\text{theory}}. \tag{S42}$$

This theoretical relationship is displayed in Fig. S11**a** (the yellow solid line), which is consistent with the simulation results (the blue dots). According to the simulation results, $b \approx 2.18$.

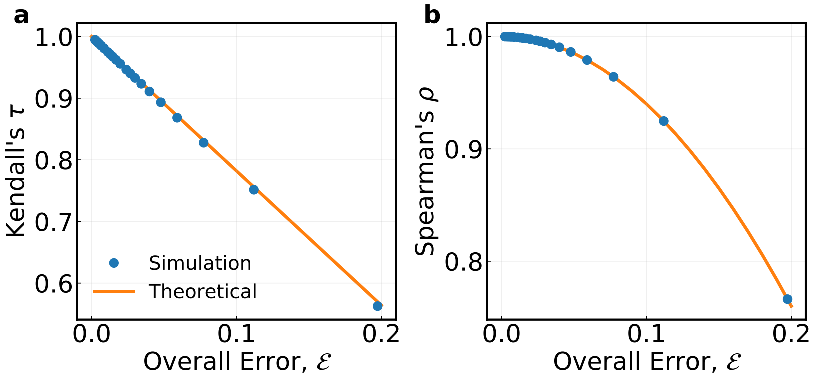

Figure S11: The relationship between the overall error $\mathcal{E}$ and **a** Kendall's τ, **b** Spearman's ρ. The yellow solid lines represent the theoretical relationships and the blue dots are the simulation results. The simulation is performed under the number of edges $E = 10000$ and the pairwise accuracy $x \in [0.51, 0.95]$. Each dot (the combination of E and x) is repeated 10 times.

6.2.2 Theoretical equivalence between $\mathcal{E}$ and Spearman's ρ.

By the definition of Spearman's ρ, we have

$$\rho = 1 - \frac{6\sum_{i=1}^{E} D_i^2}{E(E^2-1)} = 1 - \frac{6E^2}{E^2-1} \times \frac{1}{E}\sum_{i=1}^{D}\left(\frac{D_i}{E}\right)^2 = 1 - \frac{6E^2}{E^2-1}\mathcal{E}^2. \tag{S43}$$

When E is large, we have $E^2 \approx E^2 - 1$ so that

$$\rho = 1 - 6\mathcal{E}^2. \tag{S44}$$

This theoretical relationship is displayed in Fig. S11**b** (the yellow solid line), which is also consistent with the simulation results (the blue dots)

7 Discussion of restoration results for networks lacking ground truth

The case of "lacking ground truth data" is defined as: we do not have the fine-grained ground truth of the order in which each edge in the network is generated; only the ground truth of the coarse-grained snapshots of the edges are available. For networks lacking ground truth, for example, PPI networks, with only 2-4 snapshots, the restoration results of edges within the same snapshot cannot be effectively

verified. This is a generic difficult problem for many machine learning techniques. Here we provide a preliminary discussion on this topic.

Due to the randomness of the restoration model itself, (including the embedding and the training of the ensemble model), the results will be different every time we go through the restoration process. This inspires us to verify the credibility of the restoration by "cross-validation" on these random results.

The idea of "cross-validation" is based on the following intuitive assumption: if the restoration process is reliable, then the random results should all be close to each other since they are all close to the ground truth; however, if the restoration process is unreliable, then the random results should be far away from each other since they are far away from the ground truth in different directions. Based on such an intuitive assumption, we could verify the credibility of the restoration by examining the difference between the random results. Specifically:

1). Examine the difference between results from multiple trainings using the same machine learning model. We find that as more edge pairs are used to train the model, the outputs from the same model become closer (Fig. S12a-c).

2). Examine the difference between the results of different machine learning models. We find that as more edge pairs are used to train the model, the outputs of the different models also become closer (Fig. S12d-f).

Both observations indicate that the reconstruction process is reliable. Note that when measuring the difference between two restored edge sequences, the error $\mathcal{E}$ defined by Eq. (1) in our main text is used by treating one of the two restored edge sequences as the ground-truth sequence. This is the reason why the $\mathcal{E}$ in Fig. S12 and Fig. S13 are larger than those from the theoretical results in Fig. 2b of the main text. Since our approach involves comparing two random results rather than comparing each with the underlying ground truth, the error here would be approximately twice the error of the theoretical results.

However, the results based on the PPI network for Fruit Fly and Worm do not follow the same pattern, especially when comparing different models (see Fig. S12c-d). Possible reasons are that the number of edges of the PPI network for Fruit Fly and Worm are relatively small compared to other PPI networks (see Tab. S1), and the pairwise accuracy of the models trained on these two PPI networks are not as high as those on other PPI networks (see Tab. S2). Therefore, we acknowledge that the restoration results for Fruit Fly and Worm may be suboptimal.

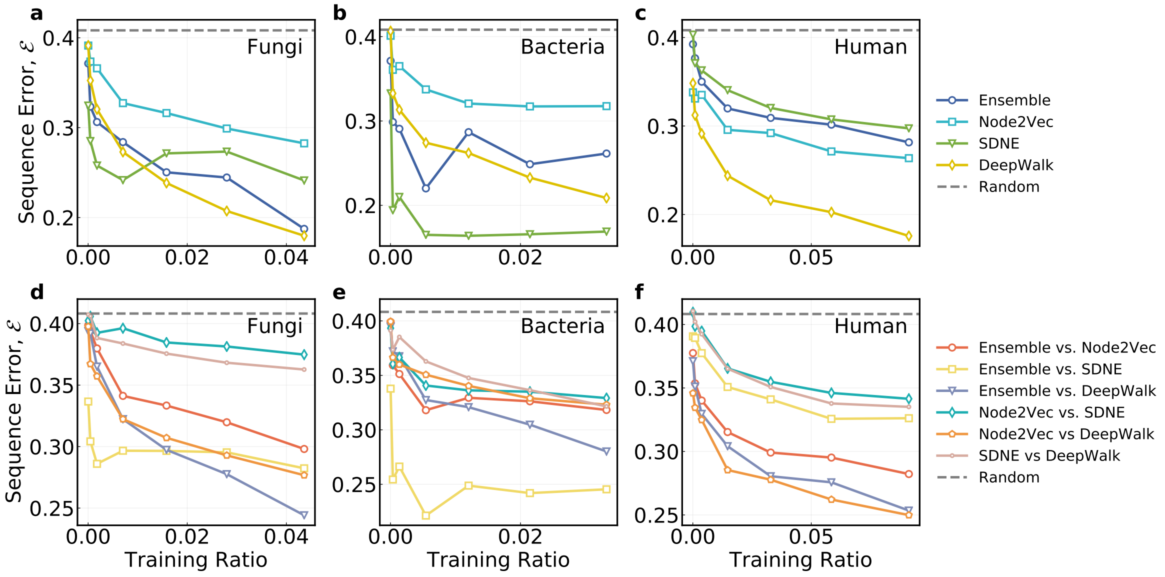

Figure S12: **Verification of the reconstruction through "cross-validation" based on the PPI network for Fungi, Bacteria, and Human. a-c** Average difference between restored edge sequences from multiple trainings of the same machine learning model. For a fixed training ratio, each machine learning model is trained 10 times to obtain 10 restored edge sequences. Then calculate the E between each pair of restored edge sequences and get 10×(10-1)/2=45 errors. Each point in **a-c** is the average of the 45 errors. **d-f** Average difference between restored edge sequences from different machine learning models (i.e., using different edge representation methods). When comparing two models, for a fixed training ratio, each model is trained 10 times so that 10 restored edge sequences are obtained for each model. Then calculate the E between each pair of restored edge sequences from the two models and get $10 \times 10 = 100$ errors. Each point in **d-f** is the average of the 100 errors. The gray dashed line represents the average difference between two random edge sequences. Please refer to Fig. S1 in the SI for an illustration of the models, i.e., the Node2Vec, DeepWalk, and SDNE correspond to the first three base models to be ensembled in Fig. S1.

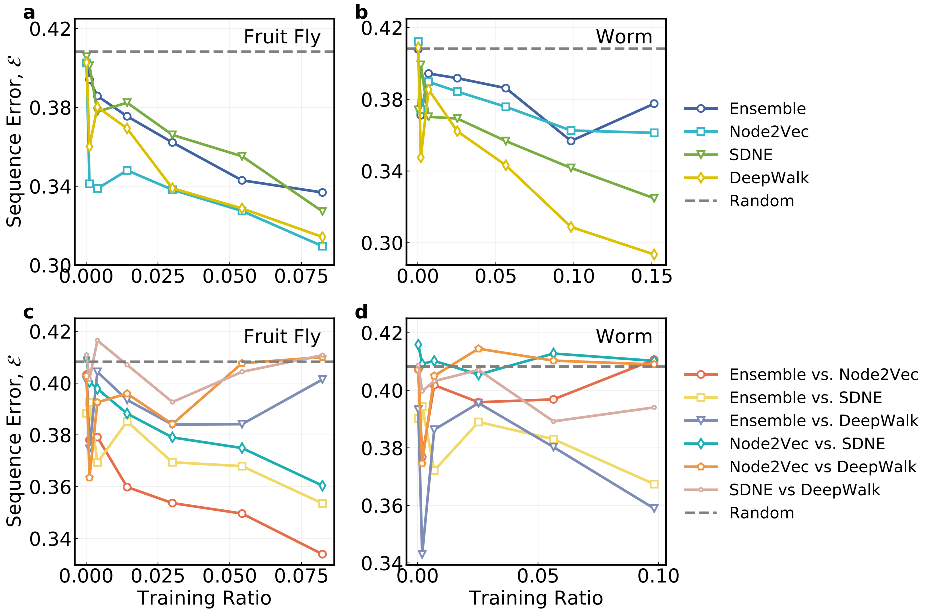

Figure S13: **Verification of the reconstruction through "cross-validation" based on the PPI network for Fruit Fly and Worm.** **a-b** Same with **a-c** in Figure S12. **c-d** Same with **d-f** in Figure S12.

8 Transfer Learning

Transfer learning in this work is to use the ensemble model trained in one network to judge the generation order of edge pairs in another network. Here we introduce the three synthetic network models we used, the details of transfer learning and the full results of transfer learning on the synthetic networks.

8.1 Synthetic network models

We consider three growing network models in this work. The details are as follows:

1. Barabási–Albert (BA) model [33]: BA modthe el is a scale-free network model based on growing and preferential attachment. As time grows, new nodes and edges are constantly added to the network, and new nodes are more like to connect to nodes with higher degrees. The construction of BA model in this work is as follows:

   (1) Generate an ER random network with the number of nodes $N_0 = 10$ and connection probability $q = 0.5$ as the initial network of the BA model.

   (2) At each time step, one new node is added to the network.

   (3) n existing nodes in the network are selected to connect with the new node added at step (2). The existing nodes are selected by the rule of preferential attachment, in which the nodes are selected with the probability proportional to the degree of the node. Mathematically, a node added at time step t connects to an existing node a with probability $P_a = \frac{k_a}{\sum_{b=1}^{N_t} k_b}$, where k_a is the degree of node a, N_t is the number of the existing nodes at time step t.

   (4) Iterate steps (2) and (3) until all nodes and edges are added.

2. Popularity-similarity-optimization (PSO) model [34,35]: PSO model is a growing network model that considers both popularity of nodes and similarity between two nodes when connecting them. The nodes of the PSO model are embedded in hyperbolic space. At each time step t, add one new node to the hyperbolic space, the radial distance of the new node is $r = \ln t$ and the angular position is random. Then n existing nodes with the smallest $s\theta_{st}$ are selected to connect with the new node, where s is the birth time of the existing node, θ_{st} is the angular distance between the new node and the existing node. Following the above rules, the radial distance of nodes in the constructed network represents their popularity, and the angular distance between nodes represents their angular distance. For implementation, we directly use the code published by the author [35]. The parameters of the code are set to: $m = 5, L = 5, \gamma = 2.1, T = 0.4, \zeta = 1$.

3. Fitness model [36]: The fitness model is a growing model based on the BA model. The idea of the fitness model is that the ability of a node to attract new edges is not only related to its degree, but also related to its inner characteristics. For example, in a social network, even though

some people join late, they can quickly make more friends with their good personalities. If the characteristics of a node are represented by fitness η, then in the construction of fitness model, all other steps are the same with BA model except that the probability of selecting existing nodes is $P_a = \frac{\eta_a k_a}{\sum_{b=1}^{N_t} \eta_b k_b}$ at step (3), in which η_a and η_b is the fitness of node a and b. The value of fitness for each node is randomly assigned by power law distribution $P(\eta) = \eta^{-3.5}$.

For each network model, we record the generation time of all edges, and the edges that are added at the same time step are not distinguishable. We generate two networks with different sizes for each model. The basic information of the six synthetic networks is shown in Tab S6.

Table S6: **Basic information of the synthetic networks.** From left to right, we report: the network name, the number of nodes N, the number of edges E, the number of edge pairs that can distinguish the generation order E_d, the ratio of the order-distinguishable edge pairs to the total number of edge pairs P_{E_d} (Note that $P_{E_d} < 1$ because the sequence of edges generated at the same step cannot be distinguished), and the number of time steps (snapshots) in a network S.

Network name	N	E	E_d	P_{E_d}	S
BA1	500	4,920	12,078,500	0.998	491
BA2	1,000	4,975	12,362,625	0.999	991
PSO1	500	3,911	7,629,866	0.998	500
PSO2	1,000	8,201	33,585,814	0.999	1,000
Fitness1	500	4,924	12,098,100	0.998	491
Fitness2	1,000	9,920	49,153,500	0.999	991

8.2 Vector transformation of nodes in transfer learning

The core of transfer learning is to quantify the similarity between the training network A and the test network B. Denote the matrices consisting of nodes' vectors in network A and B as $\mathbf{H_A}$ and $\mathbf{H_B}$, respectively. To find the similarity between $\mathbf{H_A}$ and $\mathbf{H_B}$ is to find a transformation matrix $\mathbf{L}$ such that $||\mathbf{H_B L} - \mathbf{H_A}||$ is minimized. Define the objective function as follows:

$$\begin{aligned} J(\mathbf{L}) &= ||\mathbf{H_B L} - \mathbf{H_A}||^2 \\ &= (\mathbf{H_B L} - \mathbf{H_A})^T(\mathbf{H_B L} - \mathbf{H_A}) \\ &= \mathbf{L}^T\mathbf{H_B^T H_B L} - \mathbf{L}^T\mathbf{H_B^T H_A} - \mathbf{H_A^T H_B L} + \mathbf{H_A^T H_A}. \end{aligned} \tag{S45}$$

It can be seen that minimizing $||\mathbf{H_B L} - \mathbf{H_A}||$ is equal to minimizing the function $J(\mathbf{L})$. And the minimum value of the function $J(\mathbf{L})$ is where its derivative is 0. Therefore, we take the derivative of

Eq. (S45) and let its derivative equals to 0:

$$\frac{\partial J(\mathbf{L})}{\partial \mathbf{L}} = 2\mathbf{H}_\mathbf{B}^T\mathbf{H}_\mathbf{B}\mathbf{L} - 2\mathbf{H}_\mathbf{B}^T\mathbf{H}_\mathbf{A} = 0. \tag{S46}$$

Then the transformation matrix can be obtained:

$$\mathbf{L} = (\mathbf{H}_\mathbf{B}^T\mathbf{H}_\mathbf{B})^{-1}\mathbf{H}_\mathbf{B}^T\mathbf{H}_\mathbf{A}. \tag{S47}$$

Once we have $\mathbf{L}$, we can use it to get the transformed matrix $\mathbf{H}_\mathbf{B}^{'}$ of the nodes in the test network B:

$$\mathbf{H}_\mathbf{B}^{'} = \mathbf{H}_\mathbf{B}\mathbf{L}. \tag{S48}$$

In the above transformation, the sizes of matrix $\mathbf{H}_\mathbf{A}$ and $\mathbf{H}_\mathbf{B}$ are $(N_A \times d)$ and $(N_B \times d)$, respectively, where N_A, N_B are the number of nodes in networks A and B and d is dimension of nodes' vector, which is same for nodes in different networks. However, due to the rule of matrix multiplication, $N_A = N_B$ is also required. For networks with different numbers of nodes, we adjust $\mathbf{H}_\mathbf{A}$ to make it the same dimension as $\mathbf{H}_\mathbf{B}$. The rules to adjust $\mathbf{H}_\mathbf{A}$ are introduced as follows.

First, sorting the nodes in training network A and test network B in descending order of degree. Suppose $E_A^1, ..., E_A^i, ..., E_A^{N_A}$ and $E_B^1, ..., E_B^i, ..., E_B^{N_B}$ are the vector representation of the i-th node in networks A and B after sorting, respectively. Then different ways are used in different cases.

1. When $N_A > N_B$, matches the i-th node in network B with the $\lfloor N_A/N_B \rfloor i$-th node in network A, and then take vectors of nodes in A that have matching relationship with nodes in B, i.e., $E_A^{\lfloor N_A/N_B \rfloor i}$, to form matrix $\mathbf{E'}_\mathbf{A}$ to calculate instead of $\mathbf{E}_\mathbf{A}$. Figure. S14a shows an example of adjusting matrix $\mathbf{H}_\mathbf{A}$. When $N_A = 5$, $N_B = 2$, we have $\lfloor N_A/N_B \rfloor = 2$, so E_B^1 and E_B^2 correspond to E_A^2 and E_A^4, respectively. Then E_A^2 and E_A^4 form the matrix $\mathbf{E'}_\mathbf{A}$.

2. When $N_A < N_B$, denote the integer part of N_B/N_A as a and the remainder part as b, then copy each vector of the first $N_A - b$ nodes in network A a times, and copy each vector of the last b nodes $a+1$ times, the copied vectors are used to form a matrix $\mathbf{E'}_\mathbf{A}$ to calculate instead of $\mathbf{E}_\mathbf{A}$. Figure. S14b shows an example. If $N_A = 3$, $N_B = 5$, the integer part of N_B/N_A is 1 and remainder part is 2. So E_A^1 is copied once, E_A^2 and E_A^3 are copied twice, respectively. The copied vectors $(E_A^1, E_A^2, E_A^2, E_A^3, E_A^3)^T$ form the matrix $\mathbf{E'}_\mathbf{A}$.

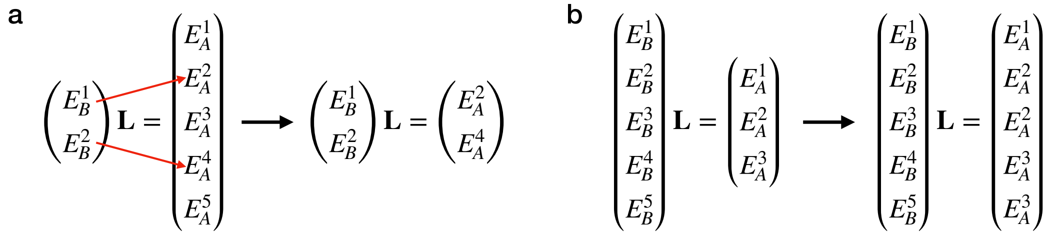

Figure S14: **Examples of aligning nodes' vectors of two networks a**. The adjustment of $\mathbf{H_A}$ when $N_A > N_B$. In the example, $N_A = 5$, $N_B = 2$, so E_B^1 and E_B^2 correspond to E_A^2 and E_A^4, respectively. Then the matrix $\mathbf{E'_A}$ is formed by E_A^2 and E_A^4. **b**. The adjustment of $\mathbf{H_A}$ when $N_A > N_B$. In the example, $N_A = 3$, $N_B = 5$. The integer part of N_B/N_A is 1 and the remainder part is 2. So E_A^1 is copied once, E_A^2 and E_A^3 are copied twice, respectively. The matrix $\mathbf{E'_A}$ is formed by the copied vectors $(E_A^1, E_A^2, E_A^2, E_A^3, E_A^3)^T$.

9 Revealing the evolution mechanisms by the restored edge sequence

9.1 Preferential attachment in real-world networks

In this section, we give details of calculating preferential attachment (PA) function $\Pi(k)$ for our real-world network data, and show cumulative PA function $\kappa(k)$ obtained by real sequence and the sequence restored by our method on networks not shown in the main text (see Fig. S15 and Fig. S16).

The PA function $\Pi(k)$ is a rate at which a node with degree k acquires new edges. When PA is present in a network, $\Pi(k)$ is positively correlated with k. When calculating it in a real-world network, consider a time interval $[T_0, T_0 + \Delta T]$ in the network evolution process, the average degree increase of nodes with degree k is the PA function $\Pi(k)$ given a small ΔT. However, due to the granularity of the network generation processes, a small ΔT would yield sparse data points causing large fluctuations of $\Pi(k)$. As a remedy, we choose ΔT values that are large enough to produce reasonable estimations and calculate the cumulative PA function $\kappa(k) = \int_1^k \Pi(k')\mathrm{d}k'$ instead [37, 38]. If the growth of a network follows the preferential attachment rule, we expect that $\kappa(k) \propto k^{\beta+1}$, where β is a positive scaling exponent. Since $\beta > 0$, $\kappa(k)$ should be a straight line with a slope greater than 1 in a log-log plot. For each network, the time interval we select is listed in Table S7.

Figure S15 gives results of all the real-world networks we use in this work. It is shown that PA is present for most of the networks considered. And even for networks without a clear pattern of PA, the results from the restored processes also agree very well with the ground truth. It is really interesting to see that the animal network (ants) in Fig. S15, where $\Pi(k)$ is shown instead of $\kappa(k)$, demonstrates an anti-PA pattern, i.e., the rate at which a node acquires new edges is negatively correlated with its degree.

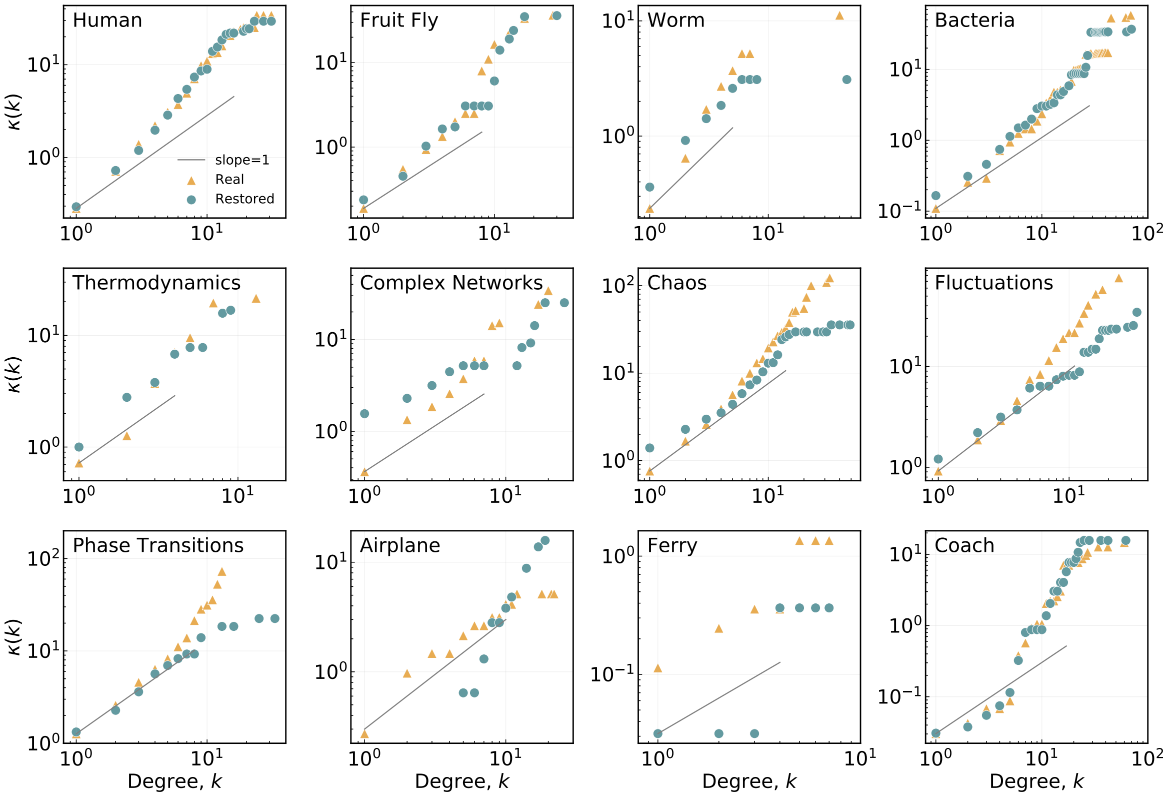

Figure S15: **Cumulative preferential attachment function $\kappa(k)$ in real-world networks.** The yellow circles and blue triangles are $\kappa(k)$ based on real and restored network evolution respectively. The gray solid lines represent the slope when preferential attachment is absent (slope = 1), the slope will be larger than 1 if network growth follows preferential attachment.

9.2 Protein categories: the abbreviations and the corresponding functions

Clusters of Orthologous Groups (COGs) are functional classifications of proteins based on their evolutionary relationships. Each protein can be classified into one COG category, we visualize the PPI network by coloring different proteins based on their functional categories in Fig. 4 and construct a function network by treating all proteins with the same function as one super-node in Fig. 5 in the main text. In this section, we list details of the abbreviations and the corresponding functions by COGs (see Tab. S8).

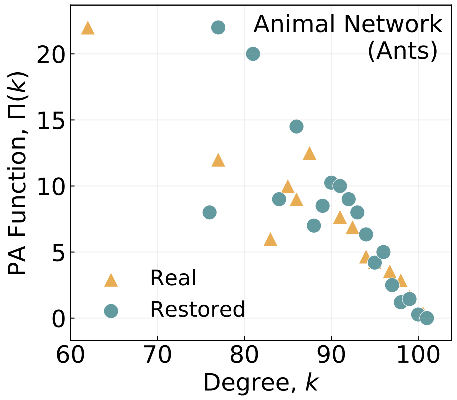

Figure S16: **Preferential attachment function $\Pi(k)$ for ants network.** The yellow circles and blue triangles are $\Pi(k)$ based on real and restored network evolution respectively.

9.3 Methods to generate network based on preferential attachment (PA) rule

In the main text, for comparison, we generated networks based on the first 100 edges of the restored edge sequence following the preferential attachment (PA) rule for different real-world networks. Next, we provide a detailed description of the network generation process.

1. Based on our full restored edge sequence, we determine the order of nodes added to the network. If an edge introduces two new nodes simultaneously, the adding order of these two nodes is randomly assigned.

2. Constructing an initial network using the first 100 edges extracted from the restored sequence.

3. Adding nodes one by one according to the order determined in step 1. For each node (including nodes that already existed in the initial network), select m existing nodes to connect. The number of newly added edges m is determined as follows:

$$m = \begin{cases} \mathrm{int}(\frac{k_{\mathrm{real}} - k_{\mathrm{existed}}}{2}), & \text{if} \quad k_{\mathrm{real}} > 1 \\ 1, & \text{if} \quad k_{\mathrm{real}} = 1 \end{cases} \tag{S49}$$

where k_{real} is the degree of node in the real network, k_{existed} is the degree of node if the node already existed in the current network ($k_{\mathrm{existed}} > 0$ only when the node is already existed in the initial network in step 2), n_{existed} is the number of node of the current network. Note that m cannot be larger than $n_{\mathrm{existed}} - k_{\mathrm{existed}} - 1$, which is the number of all nodes that are not connecting with the newly added node.

Table S7: **The selection of time interval to calculate preferential function.** From left to right, we report: the network type, network name, the number of snapshots in a network S, the initial snapshot T_0, the number of nodes in the initial snapshot N_0, the number of newly added edges ΔE in the time interval $T_0, T_0 + \Delta T$. For convenience, the final snapshot for calculating $\Gamma(k)$ is set to the actual final snapshot of each network. As for our restored sequence, the time interval $T_0, T_0 + \Delta T$ is selected ensuring that the final snapshot and the newly added edges ΔE are the same as those selected for the real sequence. Note that most newly added edges are connecting to other newly added nodes in the Weaver network, so that the degree increase of the existing nodes is too small to calculate. The results of the Weaver network are not included in the following figure.

Network type	Network name	S	T_0	N_0	ΔE
Protein-Protein Interaction (PPI)	Fungi	3	1	2,051	575
	Human	3	1	1,628	665
	Fruit Fly	3	1	406	122
	Worm	4	1	291	198
	Bacteria	2	1	777	166
World Trade Web	WTW	17	1	179	620
Collaboration	Complex Networks	172	90	146	183
	Chaos	1,118	500	1,227	1878
	Fluctuations	731	300	602	1316
	Interfaces	1,040	700	2,213	1822
	Phase Transitions	654	200	391	1383
	Thermodynamics	131	40	52	170
Animal	Weaver	8	7	419	119
	Ants	6	1	102	166
Transportation	Airplane	5	1	47	11
	Ferry	6	1	203	20
	Coach	4	1	1873	32

4. After completing step 3, we compare the number of edges in the generated network with the real network. Then randomly add or remove edges to maintain a consistent total number of edges with the real network.

Table S8: **Clusters of Orthologous Groups (COGs) Categories.** From left to right, we report the abbreviation and the corresponding function descriptions.

Abbreviation	Protein function
J	Translation, ribosomal structure and biogenesis
A	RNA processing and modification
K	Transcription
L	Replication, recombination and repair
B	Chromatin structure and dynamics
D	Cell cycle control, cell division, chromosome partitioning
Y	Nuclear structure
V	Defense mechanisms
T	Signal transduction mechanisms
M	Cell wall/membrane/envelope biogenesis
N	Cell motility
Z	Cytoskeleton
W	Extracellular structures
U	Intracellular trafficking, secretion, and vesicular transport
O	Posttranslational modification, protein turnover, chaperones
C	Energy production and conversion
G	Carbohydrate transport and metabolism
E	Amino acid transport and metabolism
F	Nucleotide transport and metabolism
H	Coenzyme transport and metabolism
I	Lipid transport and metabolism
P	Inorganic ion transport and metabolism
Q	Secondary metabolites biosynthesis, transport and catabolism
R	General function prediction only
S	Function unknown

9.4 Other network characterizations revealed by the restored edge sequence

In this section, we show the full results of the evolution of network characterizations, for example, the assortativity coefficient in Fig. S17, average local clustering coefficient in Fig. S18, and average shortest path length in Fig. S19. Table S9 gives the value of assortativity, average local clustering, and average shortest path length for all the real-world networks in the final state. Note that due to the presence of disconnected components during the evolution process of a network, the computation of the average shortest path length only involves pairs of nodes that can be connected.

Table S9: **The network characterizations in the final state of real-world networks.** From left to right, we report: the network type, network name, the degree assortativity (r) in the final snapshot, the average local clustering coefficient ($\langle C \rangle$) in the final snapshot, and the average shortest path length in the final snapshot.

Network type	Network name	Assortativity, r	Local Clustering, $\langle C \rangle$	Shortest Path Length
Protein-Protein Interaction (PPI)	Fungi	0.458	0.379	6.776
	Human	0.029	0.155	6.351
	Fruit Fly	0.068	0.178	5.885
	Worm	-0.124	0.032	7.694
	Bacteria	0.265	0.249	4.589
World Trade Web	WTW	-0.660	0.797	1.816
Collaboration	Complex Networks	-0.112	0.521	9.023
	Chaos	-0.072	0.528	9.297
	Fluctuations	-0.092	0.508	8.837
	Interfaces	0.027	0.647	7.961
	Phase Transitions	-0.075	0.488	12.548
	Thermodynamics	-0.266	0.412	7.835
Animal	Weaver	0.199	0.669	3.643
	Ants	-0.039	0.99	1.012
Transportation	Airplane	-0.352	0.337	2.361
	Ferry	0.293	0.207	2.67
	Coach	0.197	0.106	17.995

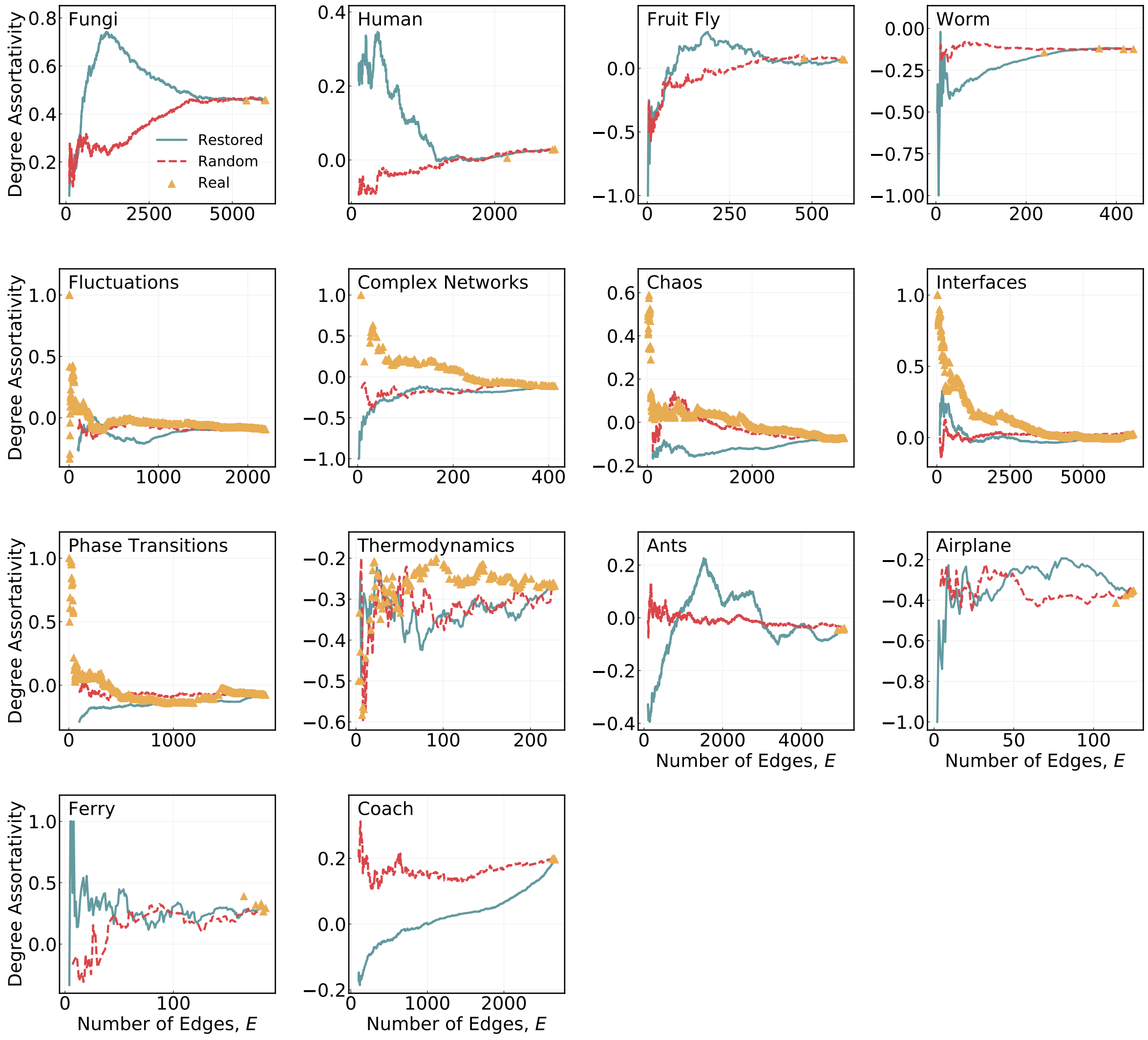

Figure S17: **Degree assortativity for real-world networks.** The yellow triangles, blue solid lines, and red dashed lines are results based on real edge generation order, edge generation order restored by our method and by random assignment, respectively.

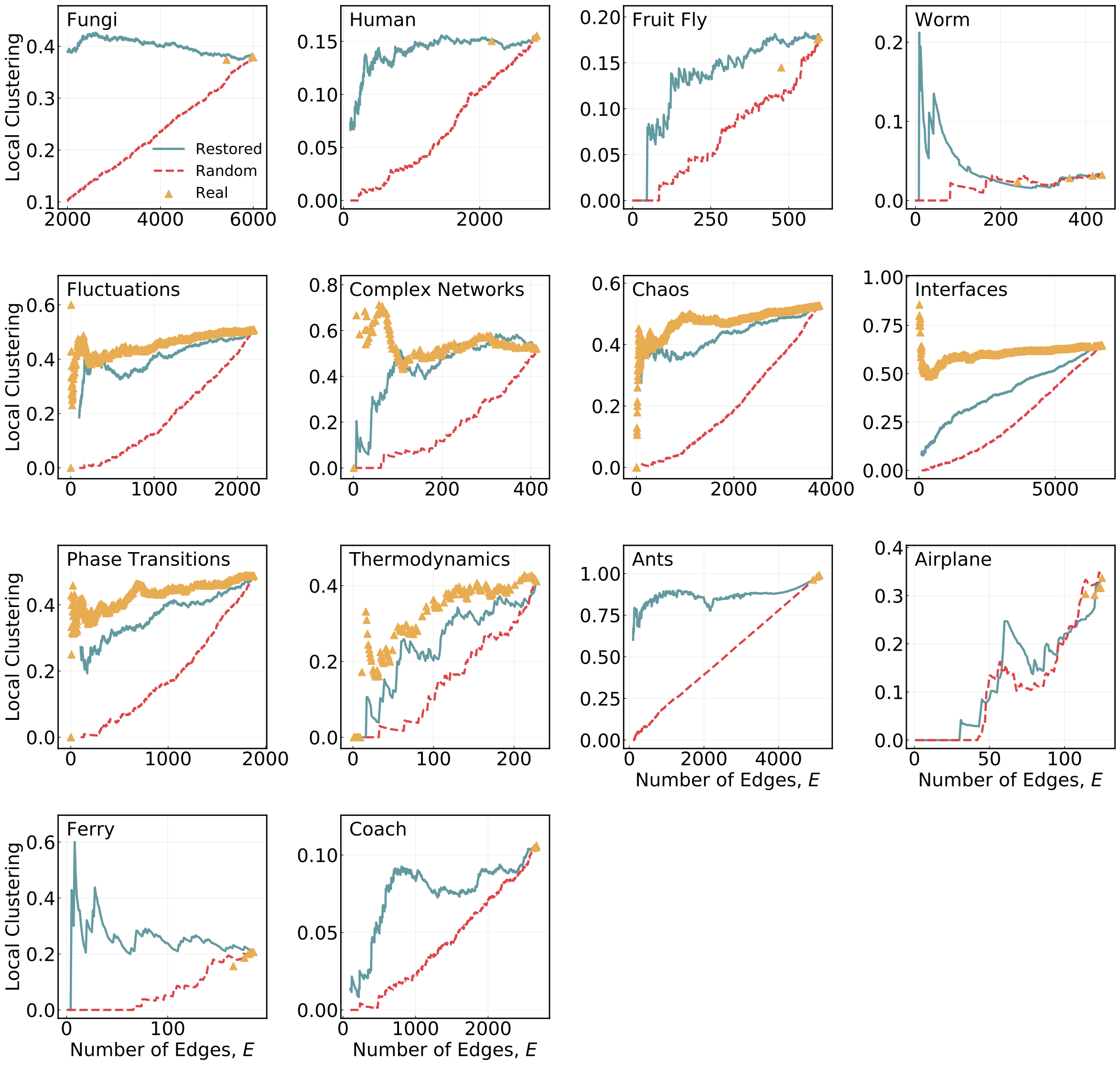

Figure S18: **Average local clustering coefficient for real-world networks.** The yellow triangles, blue solid lines, and red dashed lines are results based on real edge generation order, edge generation order restored by our method and by random assignment, respectively.

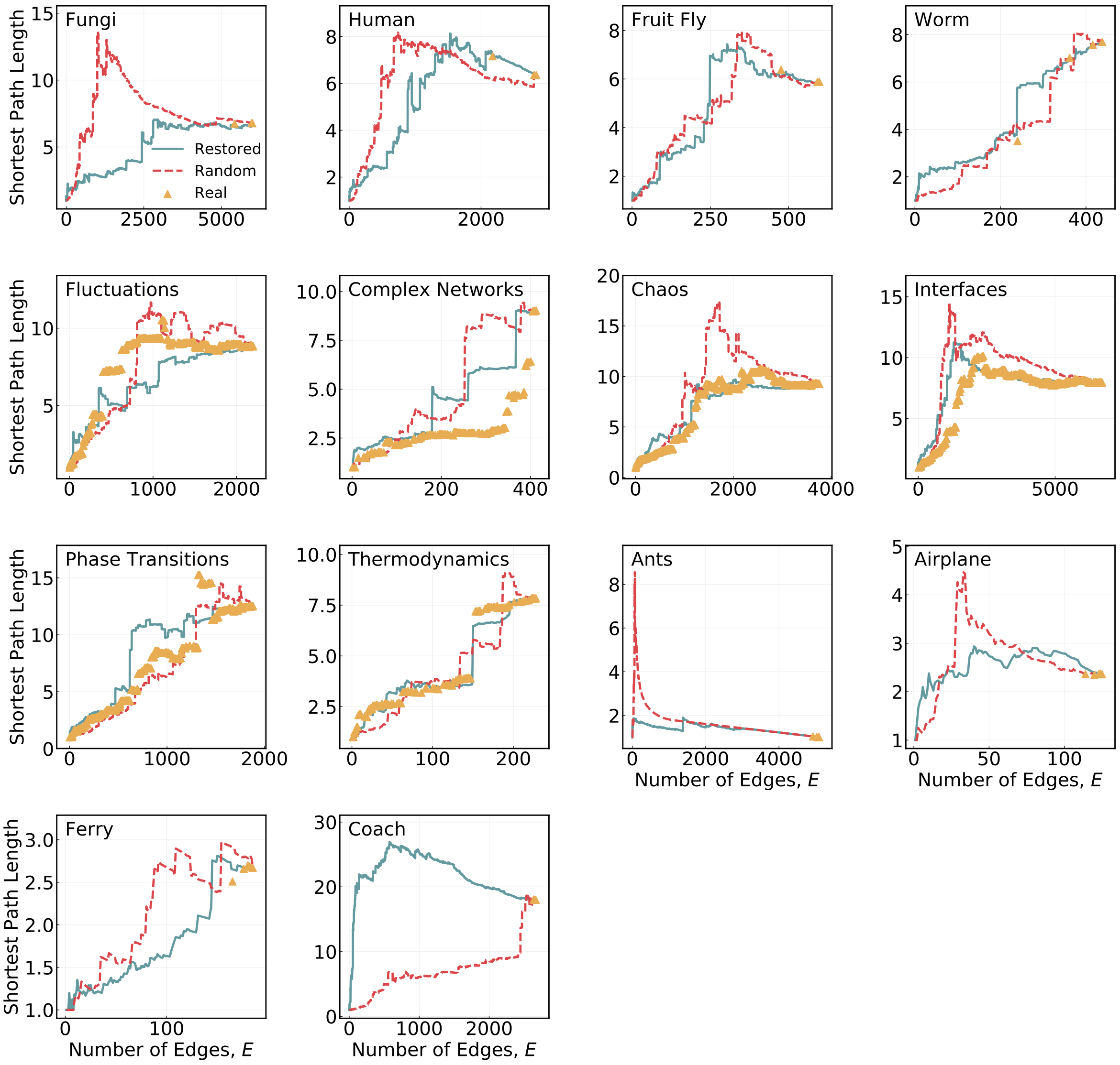

Figure S19: **Average shortest path length for real-world networks.** The yellow triangles, blue solid lines, and red dashed lines are results based on real edge generation order, edge generation order restored by our method and by random assignment, respectively.

10 Link prediction with restored edge sequence

In this section, we first list the number of the removed edges for each real-world network, and then show more results about how our restored edge generation sequence facilitates the link prediction task.

10.1 Detailed information of removed edges for link prediction

Our restored edge sequence can be used to facilitate the ability of different algorithms to predict future possible links. In order to evaluate the performance, we remove edges added at the last few snapshots in the real network data, and then use the original link prediction methods and the methods with our restored edge sequence to test how many removed edges can be predicted successfully. For different networks, since the number of the snapshots and the number of edges in different snapshots are different, the number of edges we removed is also different. Here we list detailed information of removed edges for all real-world networks we used (see Tab. S10).

10.2 Facilitating the results of common neighbor for link prediction

Except for the truncated singular value decomposition (TSVD) link prediction algorithm we have shown in the main paper, our restored edge generation sequence can also facilitate the results of other link prediction methods. In this section, we show the comparison of the link prediction results by the original common neighbor algorithm and common neighbor with our restored sequence.

The idea of common neighbor (CN) [8] is that two nodes are more likely to be connected if they share more neighbors. Denote $\Gamma(a)$ and $\Gamma(b)$ are sets of neighbors of node a and node b, respectively. The common neighbor index between them is described as:

$$S_{ab}^{\mathrm{CN}} = |\Gamma(a) \cap \Gamma(b)|. \tag{S50}$$

Set $\widehat{\boldsymbol{\alpha}} = (\widehat{\alpha_1}, \widehat{\alpha_2}, ..., \widehat{\alpha_E})$ as the restored edge generation order, where $\widehat{\alpha}_i$ represents the generation order of edge i. Then we consider the network as a weighted network, where the weight of an edge i is decided by $\widehat{\alpha}_i$:

$$w_i = \theta^{\max(\widehat{\alpha}) - \widehat{\alpha}_i}, \tag{S51}$$

$\theta \in (0, 1)$ is a free parameter that can be tuned to control the importance of edge generation order for link prediction. The weighted common neighbor index can be defined as:

$$S_{ab}^{\mathrm{WCN}} = \sum_{z \in \Gamma(a) \cap \Gamma(b)} w_{az} + w_{bz}. \tag{S52}$$

The comparison of S_{ab}^{CN} and S_{ab}^{WCN} on different real-world networks is shown in Fig. S20. It can be seen that for most of the networks, our restored edge sequence can facilitate the performance of the CN link prediction algorithm.

Table S10: **Detailed information of removed edges for link prediction.** From left to right, we report: network type, network name, the number of snapshots in a network S, the number of snapshots that we removed S^{removed} (where 0.5 means that half of the edges in the snapshot is removed), the number of total edges E, the number of removed edges E^{removed}. Note that the edges added at the last few snapshots in the Airplane network are too few so that any link prediction algorithms we used are not capable of predicting them successfully (i.e., the number of hits is zero). Therefore, we do not show the results of the Airplane network in the next two subsections.

Network type	Network name	S	S^{removed}	E	E^{removed}
Protein-Protein Interaction (PPI)	Fungi	3	1.5	6,000	299
	Human	3	1.5	2,840	349
	Fruit Fly	3	1.5	598	64
	Worm	4	2	438	76
	Bacteria	2	0.5	2,321	83
World Trade Web	WTW	17	14	3,249	381
Collaboration	Complex Networks	172	57	413	127
	Chaos	1,118	372	3,758	1095
	Fluctuations	731	243	2,198	571
	Interfaces	1,040	346	6,718	1848
	Phase Transitions	654	218	1,882	539
	Thermodynamics	131	43	228	81
Animal	Weaver	8	4	1,332	739
	Ants	6	3	5,091	16
Transportation	Airplane	5	2	125	2
	Ferry	6	3	185	5
	Coach	4	2	2,666	8

10.3 Facilitating the results of Adamic-Adar index for link prediction

Adamic-Adar (AA) [12] is a link prediction index based on common neighbors. The contribution of common neighbors with small degrees is greater than those with large degrees. AA index is defined as:

$$S_{ab}^{\text{AA}} = \sum_{z \in \Gamma(a) \cap \Gamma(b)} \frac{1}{\log(k(z))}, \tag{S53}$$

where $k(z)$ is degree of the common neighbor z.

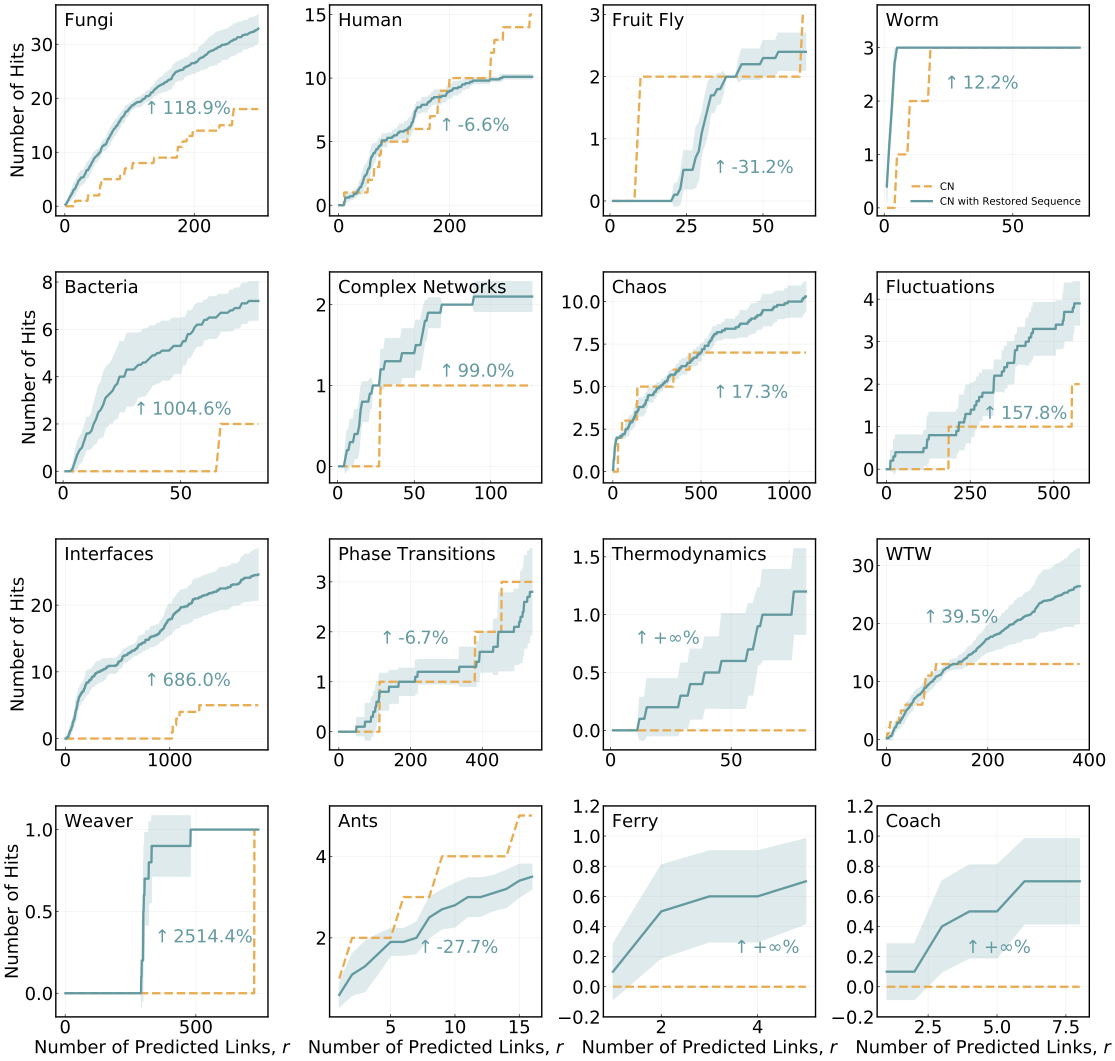

Figure S20: **Restored sequences facilitate common neighbor (CN) link prediction.** Number of hits obtained by using the CN index (yellow dashed lines) and CN index with our restored edge generation order (blue solid lines) on real-world networks. The percentage of improvement is computed based on the area under the curve. Results based on the restored sequences are averaged over 10 repeated simulations, with the light blue areas representing the 95% confidence intervals.

Same as the weighted common neighbor index, the weighted Adamic-Adar index is defined as

follows:

$$S_{ab}^{\mathrm{WAA}} = \sum_{z \in \Gamma(a) \cap \Gamma(b)} \frac{w_{az} + w_{bz}}{\log(k(z))}, \tag{S54}$$

where edge weight w_{az} and w_{bz} are defined by Eq. S51.

Figure S21 shows the comparison of S_{ab}^{AA} and S_{ab}^{WAA} on different real-world networks. It can be seen that our restored edge sequence can facilitate the performance of the AA link prediction algorithm for most networks.

10.4 Facilitating the results of structural perturbation method (SPM) for link prediction

Structural perturbation method (SPM) [39] proposed by Linyuan Lv et.al assumes that the regularity of a network can be characterized by the consistency of structural features before and after a small perturbation of network structure (e.g., random removal of a small group of links). It is calculated by:

$$S_{ab}^{\mathrm{SPM}} = \tilde{A}_{ab}, \tag{S55}$$

where $\tilde{A}$ corresponds to the perturbed matrix constructed via first-order approximation.

For SPM considering our restore edge sequence, the elements of the adjacency matrix are multiplied by weight defined in Eq. (S51) while other procedures are not changed. The comparison of SPM and SPM with restored edge sequence on different real-world networks is shown in Fig. S22. It can be seen that our restored edge sequence can facilitate the performance of the SPM link prediction algorithm.

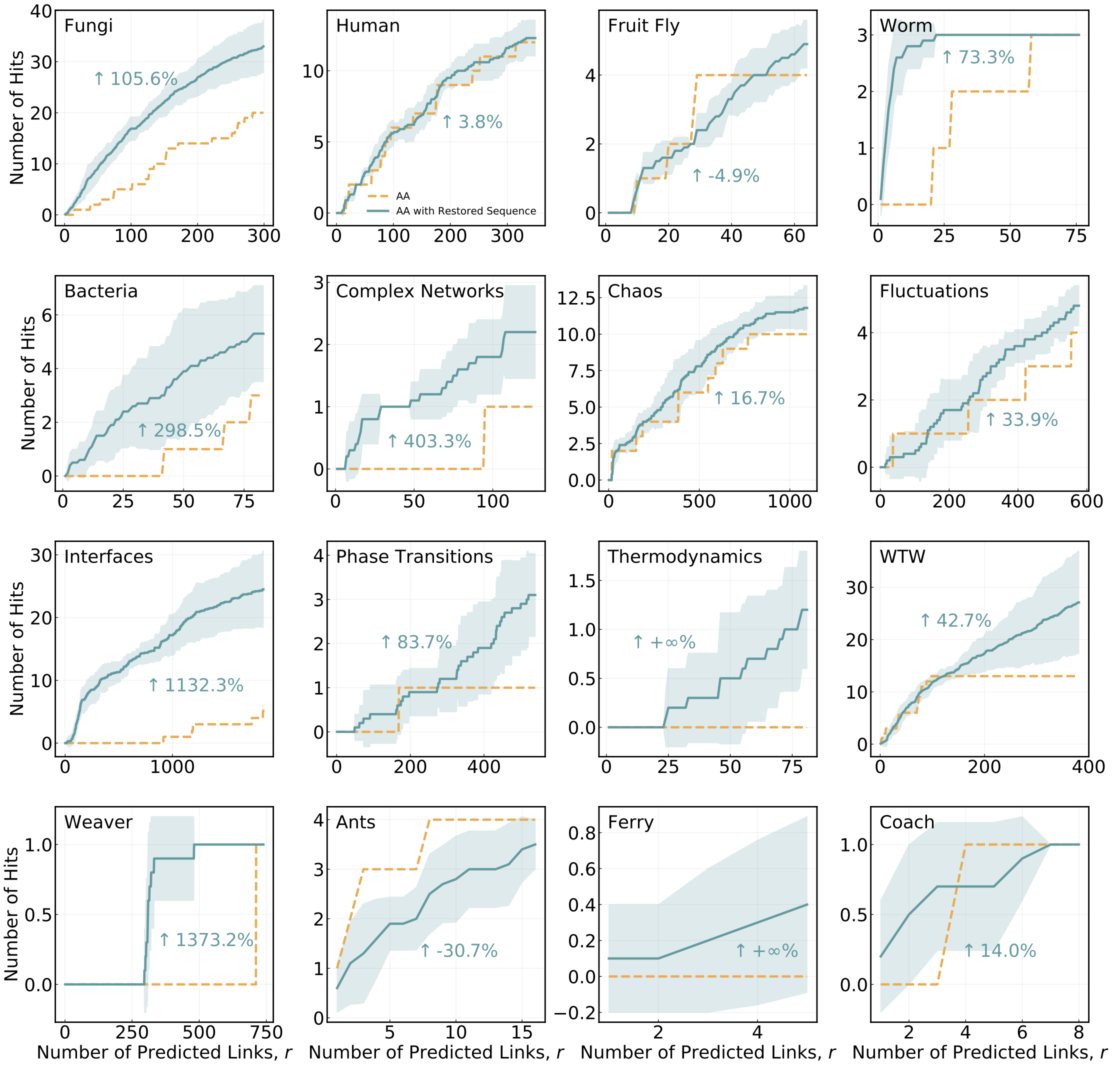

Figure S21: **Restored sequences facilitate Adamic-Adar (AA) link prediction.** The number of hits obtained by using the AA index (red dashed lines) and the AA index with our restored edge generation order (blue solid lines) on real-world networks. The percentage of improvement is computed based on the area under the curve. Results based on the restored sequences are averaged over 10 repeated simulations, with the light blue areas representing the 95% confidence intervals.

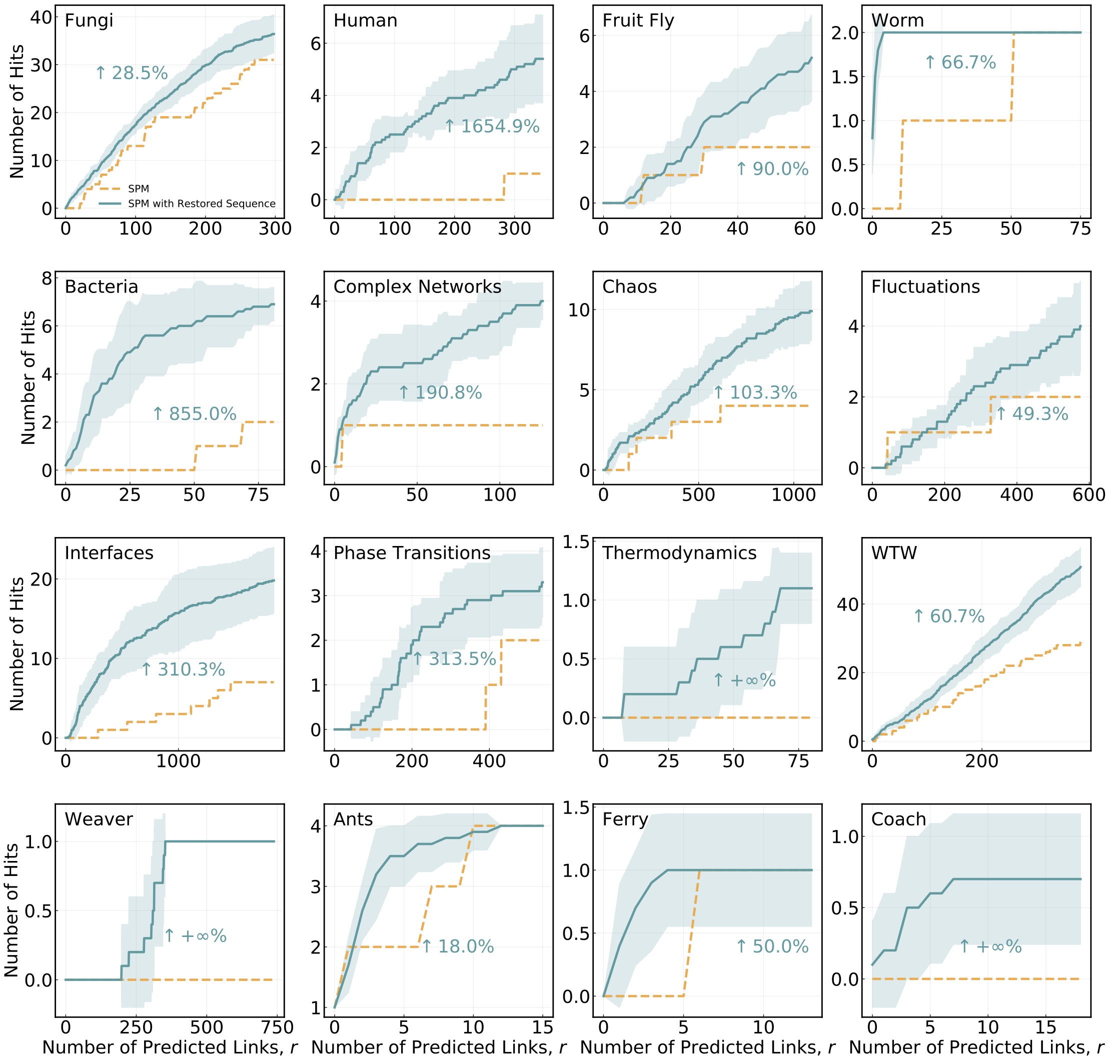

Figure S22: **Restored sequences facilitate structural perturbation method (SPM) link prediction.** Number of hits obtained by using the SPM index (yellow dashed lines) and SPM index with our restored edge generation order (blue solid lines) on real-world networks. The percentage of improvement is computed based on the area under the curve. Results based on the restored sequences are averaged over 10 repeated simulations, with the light blue areas representing the 95% confidence intervals.

Supplementary References

[1] Gerald Tesauro. Connectionist learning of expert preferences by comparison training. In *Proceedings of the 1st International Conference on Neural Information Processing Systems*, NIPS'88, page 99–106, Cambridge, MA, USA, 1988.

[2] Bryan Perozzi, Rami Al-Rfou, and Steven Skiena. Deepwalk: Online learning of social representations. In *Proceedings of the 20th ACM SIGKDD International Conference on Knowledge Discovery and Data Mining*, KDD '14, New York, NY, USA, 2014.

[3] Aditya Grover and Jure Leskovec. Node2vec: Scalable feature learning for networks. In *Proceedings of the 22nd ACM SIGKDD International Conference on Knowledge Discovery and Data Mining*, KDD '16, page 855–864, New York, NY, USA, 2016.

[4] Jian Tang, Meng Qu, Mingzhe Wang, Ming Zhang, Jun Yan, and Qiaozhu Mei. Line: Large-scale information network embedding. In *Proceedings of the 24th International Conference on World Wide Web*, WWW '15, page 1067–1077, Republic and Canton of Geneva, CHE, 2015.

[5] Leonardo F.R. Ribeiro, Pedro H.P. Saverese, and Daniel R. Figueiredo. Struc2vec: Learning node representations from structural identity. In *Proceedings of the 23rd ACM SIGKDD International Conference on Knowledge Discovery and Data Mining*, KDD '17, page 385–394, New York, NY, USA, 2017.

[6] Daixin Wang, Peng Cui, and Wenwu Zhu. Structural deep network embedding. In *Proceedings of the 22nd ACM SIGKDD International Conference on Knowledge Discovery and Data Mining*, KDD '16, page 1225–1234, New York, NY, USA, 2016.

[7] Linton C. Freeman. A set of measures of centrality based on betweenness. *Sociometry*, 40(1):35–41, 1977.

[8] David Liben-Nowell and Jon Kleinberg. The link-prediction problem for social networks. *Journal of the American Society for Information Science and Technology*, 58(7):1019–1031, 2007.

[9] Jianxin Wang, Min Li, Huan Wang, and Yi Pan. Identification of essential proteins based on edge clustering coefficient. *IEEE/ACM Transactions on Computational Biology and Bioinformatics*, 9(4):1070–1080, 2011.

[10] Mark Granovetter. The strength of weak ties. *American Journal of Sociology*, 78(6):1360–1380, 1973.

[11] Tao Zhou, Linyuan Lü, and Yi-Cheng Zhang. Predicting missing links via local information. *The European Physical Journal B*, 71(4):623–630, 2009.

[12] Lada A. Adamic and Eytan Adar. Friends and neighbors on the web. *Social Networks*, 25(3):211–230, 2003.

[13] Albert-László Barabási and Réka Albert. Emergence of scaling in random networks. *science*, 286(5439):509–512, 1999.

[14] Yan-Bo Xie, Tao Zhou, and Bing-Hong Wang. Scale-free networks without growth. *Physica A: Statistical Mechanics and its Applications*, 387(7):1683–1688, 2008.

[15] Linyuan Lü, Ci-Hang Jin, and Tao Zhou. Similarity index based on local paths for link prediction of complex networks. *Physical Review E*, 80(4):046122, 2009.

[16] Lawrence Page, Sergey Brin, Rajeev Motwani, and Terry Winograd. The pagerank citation ranking: Bringing order to the web. Technical Report 1999-66, Stanford InfoLab, 1999.

[17] Maksim Kitsak, Lazaros K. Gallos, Shlomo Havlin, Fredrik Liljeros, Lev Muchnik, H. Eugene Stanley, and Hernán A. Makse. Identification of influential spreaders in complex networks. *Nature physics*, 6(11):888–893, 2010.

[18] Saurabh Karsoliya. Approximating number of hidden layer neurons in multiple hidden layer bpnn architecture. *International Journal of Engineering Trends and Technology*, 3(6):714–717, 2012.

[19] Thomas M Cover. *Elements of information theory.* John Wiley & Sons, 1999.

[20] Diederik P. Kingma and Jimmy Ba. Adam: A method for stochastic optimization. In *3rd International Conference on Learning Representations*, San Diego, CA, USA, 2015.

[21] Peter Emerson. The original borda count and partial voting. *Social Choice and Welfare*, 40(2):353–358, 2013.

[22] Yuliang Jin, Dmitrij Turaev, Thomas Weinmaier, Thomas Rattei, and Hernán A. Makse. The evolutionary dynamics of protein-protein interaction networks inferred from the reconstruction of ancient networks. *Plos One*, 8(3):1–15, 2013.

[23] Guillermo García-Pérez, Marián Boguñá, Antoine Allard, and M. Ángeles Serrano. The hidden hyperbolic geometry of international trade: World trade atlas 1870–2013. *Scientific Reports*, 6(1):1–10, 2016.

[24] Yanqing Hu, Shlomo Havlin, and Hernán A. Makse. Conditions for viral influence spreading through multiplex correlated social networks. *Physical Review X*, 4(2):021031, 2014.

[25] Ryan A. Rossi and Nesreen K. Ahmed. The network data repository with interactive graph analytics and visualization. In *Proceedings of the Twenty-Ninth AAAI Conference on Artificial Intelligence*, AAAI'15, page 4292–4293, 2015.

[26] Riccardo Gallotti and Marc Barthelemy. The multilayer temporal network of public transport in Great Britain. *Scientific Data*, 2(1):1–8, 2015.

[27] Haiyuan Yu, Pascal Braun, Muhammed A. Yıldırım, et al. High-quality binary protein interaction map of the yeast interactome network. *Science*, 322(5898):104–110, 2008.

[28] Jean-François Rual, Kavitha Venkatesan, Tong Hao, Tomoko Hirozane-Kishikawa, et al. Towards a proteome-scale map of the human protein–protein interaction network. *Nature*, 437(7062):1173–1178, 2005.

[29] René E. van Dijk, Jennifer C. Kaden, Araceli Argüelles-Ticó, Deborah A. Dawson, Terry Burke, and Ben J. Hatchwell. Cooperative investment in public goods is kin directed in communal nests of social birds. *Ecology Letters*, 17(9):1141–1148, 2014.

[30] Danielle P. Mersch, Alessandro Crespi, and Laurent Keller. Tracking individuals shows spatial fidelity is a key regulator of ant social organization. *Science*, 340(6136):1090–1093, 2013.

[31] Maurice G. Kendall. A new measure of rank correlation. *Biometrika*, 30(1/2):81–93, 1938.

[32] Charles Spearman. The proof and measurement of association between two things. *The American Journal of Psychology*, 15(1):72–101, 1961.

[33] Albert-László Barabási, Réka Albert, and Hawoong Jeong. Mean-field theory for scale-free random networks. *Physica A: Statistical Mechanics and its Applications*, 272(1):173–187, 1999.

[34] Fragkiskos Papadopoulos, Maksim Kitsak, M. Ángeles Serrano, Marián Boguná, and Dmitri Krioukov. Popularity versus similarity in growing networks. *Nature*, 489(7417):537–540, 2012.

[35] Fragkiskos Papadopoulos, Constantinos Psomas, and Dmitri Krioukov. Network mapping by replaying hyperbolic growth. *IEEE/ACM Transactions on Networking*, 23(1):198–211, 2015.

[36] Ginestra Bianconi and Albert-László Barabási. Competition and multiscaling in evolving networks. *Europhysics Letters*, 54(4):436–442, 2001.

[37] Albert-László Barabási, H Jeong, Z Néda, E Ravasz, A Schubert, and T Vicsek. Evolution of the social network of scientific collaborations. *Physica A: Statistical Mechanics and its Applications*, 311(3):590–614, 2002.

[38] H Jeong, Z Néda, and Albert-László Barabási. Measuring preferential attachment in evolving networks. *Europhysics Letters*, 61(4):567–572, 2003.

[39] Linyuan Lü, Liming Pan, Tao Zhou, Yi-Cheng Zhang, and H. Eugene Stanley. Toward link predictability of complex networks. *Proceedings of the National Academy of Sciences*, 112(8):2325–2330, 2015.
