# Supplementary material for: Reconstructing the evolution history of networked complex systems
Source: Nat Commun. 2024 Apr 2;15:2849. doi: 10.1038/s41467-024-47248-x (PMC10987487; doi:10.1038/s41467-024-47248-x)
Supplement: Supplementary file 3 — Description of Additional Supplementary Files [file 41467_2024_47248_MOESM3_ESM.pdf]

Description of Additional Supplementary Files

Supplementary Movie 1: Comparison of restored and random formation processes on PPI (fungi) network.

Supplementary Movie 2: Comparison of restored and random formation processes on PPI (worm) network.

Supplementary Movie 3: Comparison of restored and random formation processes on collaboration (complex networks) network.
