# Supplementary material for: Reconstructing the evolution history of networked complex systems
Source: Nat Commun. 2024 Apr 2;15:2849. doi: 10.1038/s41467-024-47248-x (PMC10987487; doi:10.1038/s41467-024-47248-x)
Supplement: Supplementary file 7 — Reporting Summary [file 41467_2024_47248_MOESM7_ESM.pdf]

nature portfolio

Corresponding author(s): Yanqing Hu

Last updated by author(s): Jan 4, 2024

Reporting Summary

Nature Portfolio wishes to improve the reproducibility of the work that we publish. This form provides structure for consistency and transparency in reporting. For further information on Nature Portfolio policies, see our Editorial Policies and the Editorial Policy Checklist.

Statistics

For all statistical analyses, confirm that the following items are present in the figure legend, table legend, main text, or Methods section.

n/a	Confirmed	
☐	☒	The exact sample size (*n*) for each experimental group/condition, given as a discrete number and unit of measurement
☒	☐	A statement on whether measurements were taken from distinct samples or whether the same sample was measured repeatedly
☒	☐	The statistical test(s) used AND whether they are one- or two-sided<br>*Only common tests should be described solely by name; describe more complex techniques in the Methods section.*
☒	☐	A description of all covariates tested
☒	☐	A description of any assumptions or corrections, such as tests of normality and adjustment for multiple comparisons
☐	☒	A full description of the statistical parameters including central tendency (e.g. means) or other basic estimates (e.g. regression coefficient) AND variation (e.g. standard deviation) or associated estimates of uncertainty (e.g. confidence intervals)
☒	☐	For null hypothesis testing, the test statistic (e.g. *F*, *t*, *r*) with confidence intervals, effect sizes, degrees of freedom and *P* value noted<br>*Give P values as exact values whenever suitable.*
☒	☐	For Bayesian analysis, information on the choice of priors and Markov chain Monte Carlo settings
☒	☐	For hierarchical and complex designs, identification of the appropriate level for tests and full reporting of outcomes
☒	☐	Estimates of effect sizes (e.g. Cohen's *d*, Pearson's *r*), indicating how they were calculated

Our web collection on statistics for biologists contains articles on many of the points above.

Software and code

Policy information about availability of computer code

Data collection	All data used in this study were downloaded directly from public web pages. No software was used to collect data.
Data analysis	Python (version 3.6) was used for all data analysis in this study. The code to perform data analysis are available at https://github.com/yijiaozhang/evolution_restore.

For manuscripts utilizing custom algorithms or software that are central to the research but not yet described in published literature, software must be made available to editors and reviewers. We strongly encourage code deposition in a community repository (e.g. GitHub). See the Nature Portfolio guidelines for submitting code & software for further information.

Data

Policy information about availability of data

All manuscripts must include a data availability statement. This statement should provide the following information, where applicable:

- Accession codes, unique identifiers, or web links for publicly available datasets
- A description of any restrictions on data availability
- For clinical datasets or third party data, please ensure that the statement adheres to our policy

All data used in this study are available at https://github.com/yijiaozhang/evolution_restore.

Research involving human participants, their data, or biological material

Policy information about studies with human participants or human data. See also policy information about sex, gender (identity/presentation), and sexual orientation and race, ethnicity and racism.

Reporting on sex and gender	Not applicable since our study does not involve human participants.
Reporting on race, ethnicity, or other socially relevant groupings	Not applicable since our study does not involve human participants.
Population characteristics	Not applicable since our study does not involve human participants.
Recruitment	Not applicable since our study does not involve human participants.
Ethics oversight	Not applicable since our study does not involve human participants.

Note that full information on the approval of the study protocol must also be provided in the manuscript.

Field-specific reporting

Please select the one below that is the best fit for your research. If you are not sure, read the appropriate sections before making your selection.

☐ Life sciences ☐ Behavioural & social sciences ☒ Ecological, evolutionary & environmental sciences

For a reference copy of the document with all sections, see nature.com/documents/nr-reporting-summary-flat.pdf

Ecological, evolutionary & environmental sciences study design

All studies must disclose on these points even when the disclosure is negative.

Study description	Our study aims to restore the detailed evolution process of real-world networks with limited history using machine learning methods. The real-world network data used in this study are downloaded directly from public web pages provided by the original authors. No experiment (other than computer simulation) is performed to support this study.
Research sample	This study is based on data of complex networks with some evolution history downloaded directly from public web pages provided by the original authors, including protein-protein interaction (PPI) networks, World Trade Web, collaboration networks, animal interaction networks, and transportation networks.
Sampling strategy	No experiment (other than computer simulation) is performed to support this study. In terms of computer simulation, simulations are based on real-world networks (the same nodes and edges) but assuming random chronological orders of the edges. Python's random.choice() function is used to assign random orders to the edges and to randomly split the data (i.e., the edge pairs) into training and testing sets to train the machine learning models.<br>The number of nodes (N) and number of edges (E) of some real-world networks are:<br>PPI network (Fungi): N=2,144, E=6,000.<br>PPI network (Human): N=1,891, E=2,840.<br>PPI network (Fruit Fly): N=461, E=598.<br>World Trade Web: N=187, E=3,249.<br>Collaboration network (CN): N=225, E=413.<br>Animal networks (Weaver): N=445, E=1,332.<br>Animal networks (Ant): N=102, E=5,091.<br>Transportation networks (Airplane): N=48, E=125.
Data collection	All data used in this study were downloaded directly from public web pages:<br>PPI networks: https://journals.plos.org/plosone/article?id=10.1371/journal.pone.0058134<br>World Trade Web: https://www.nature.com/articles/srep33441#MOES<br>Collaboration networks: https://www.pnas.org/doi/full/10.1073/pnas.2100151119<br>Animal networks (Weaver): https://onlinelibrary.wiley.com/doi/10.1111/ele.12320<br>Animal networks (Ant): https://www.science.org/doi/10.1126/science.1234316<br>Transportation networks: https://www.nature.com/articles/sdata201456#Sec8
Timing and spatial scale	PPI networks: from ancestral proteins to present-day (the evolution processes of protein interaction networks are very very long, no specific start or end date is provided by the original authors)<br>World Trade Web: Year 1997-2013 (no specific month is provided by the original authors), world-wide<br>Collaboration networks: Year 1978-2010 (no specific month is provided by the original authors), the collaboration between authors publishing on the American Physical Society<br>Animal networks (Weaver): 10 months duration (Sep 2010 - Dec 2010, Sep 2011 - Dec 2011, Jan 2013 - Feb 2013) in Kimberley, South Africa<br>Animal networks (Ants): 41 days duration (March 2007 - April 2007) at the University of Lausanne, Switzerland

	Transportation networks: one week in October, 2010 in the UK
Data exclusions	No data were excluded from the analyses.
Reproducibility	Not applicable.
Randomization	Randomization is not relevant to this study. The "random assignment" in the manuscript refers to the random chronological order assigned to the edges of a network, not the randomization in a traditional experiment.
Blinding	Blinding is not relevant to this study, because there is no traditional experiment in this study.

Did the study involve field work? ☐ Yes ☒ No

Reporting for specific materials, systems and methods

We require information from authors about some types of materials, experimental systems and methods used in many studies. Here, indicate whether each material, system or method listed is relevant to your study. If you are not sure if a list item applies to your research, read the appropriate section before selecting a response.

Materials & experimental systems

n/a	Involved in the study	
☒	☐	Antibodies
☒	☐	Eukaryotic cell lines
☒	☐	Palaeontology and archaeology
☒	☐	Animals and other organisms
☒	☐	Clinical data
☒	☐	Dual use research of concern
☒	☐	Plants

Methods

n/a	Involved in the study	
☒	☐	ChIP-seq
☒	☐	Flow cytometry
☒	☐	MRI-based neuroimaging

Plants

Seed stocks	Not applicable since our study does not involve any plants.
Novel plant genotypes	Not applicable since our study does not involve any plants.
Authentication	Not applicable since our study does not involve any plants.
